# Detailed characterization of the mouse embryonic stem cell transcriptome reveals novel genes and intergenic splicing associated with pluripotency

**DOI:** 10.1186/1471-2164-9-155

**Published:** 2008-04-09

**Authors:** Galih Kunarso, Kee-Yew Wong, Lawrence W Stanton, Leonard Lipovich

**Affiliations:** 1 Computational & Mathematical Biology, Genome Institute of Singapore, 60 Biopolis Street #02-01, Singapore 138672, Singapore; 2 Stem Cell & Developmental Biology, Genome Institute of Singapore, 60 Biopolis Street #02-01, Singapore 138672, Singapore; 3 Department of Biological Sciences, National University of Singapore, 14 Science Drive 4, Singapore 117543, Singapore; 4 School of Computer Engineering, Nanyang Technological University, Block N4, Nanyang Avenue, Singapore 639798, Singapore; 5 Center for Molecular Medicine and Genetics & Department of Neurology, School of Medicine, Wayne State University, 3228 Scott Hall, 540 E. Canfield Street, Detroit, MI 48201, USA

## Abstract

**Background:**

Transcriptional control of embryonic stem (ES) cell pluripotency has been a subject of intense study. Transcriptional regulators including Oct4 (Oct3/4 index), Sox2 and Nanog are fundamental for maintaining the undifferentiated state. However, the ES cell transcriptome is not limited to their targets, and exhibits considerable complexity when assayed with microarray, MPSS, cDNA/EST sequencing, and SAGE technologies. To identify novel genes associated with pluripotency, we globally searched for ES transcripts not corresponding to known genes, validated their sequences, determined their expression profiles, and employed RNAi to test their function.

**Results:**

Gene Identification Signature (GIS) analysis, a SAGE derivative distinguished by paired 5' and 3' transcript end tags, identified 153 candidate novel transcriptional units (TUs) distinct from known genes in a mouse E14 ES mRNA library. We focused on 16 TUs free of artefacts and mapping discrepancies, five of which were validated by RTPCR product sequencing. Two of the TUs were revealed by annotation to represent novel protein-coding genes: a PRY-domain cluster member and a KRAB-domain zinc finger. The other three TUs represented intergenic splicing events involving adjacent, functionally unrelated protein-coding genes transcribed in the same orientation, with one event potentially encoding a fusion protein containing domains from both component genes (Clk2 and Scamp3). Expression profiling using embryonic samples and adult tissue panels confirmed that three of the TUs were unique to or most highly expressed in ES cells. Expression levels of all five TUs dropped dramatically during three distinct chemically induced differentiation treatments of ES cells in culture. However, siRNA knockdowns of the TUs did not alter mRNA levels of pluripotency or differentiation markers, and did not affect cell morphology.

**Conclusion:**

Transcriptome libraries retain considerable potential for novel gene discovery despite massive recent cDNA and EST sequencing efforts; cDNA and EST evidence for these ES cell TUs had been limited or absent. RTPCR and full-length sequencing remain essential in resolving the bottleneck between numerous candidate novel transcripts inferred from high-throughput sequencing and the small fraction that can be validated. RNAi results indicate that, despite their strong association with pluripotency, these five transcriptomic novelties may not be required for maintaining it.

## Background

Embryonic stem (ES) cells are self-renewable cells able to differentiate into virtually any cell type, an ability called pluripotency (reviewed in [1]). Besides obvious therapeutic potential, pluripotency provides an opportunity to understand how differentiation works in early embryonic development. Many groups aim to characterize the 'stemness' of ES cells in terms of gene regulation and to identify genes responsible for maintaining pluripotency. Although the identification of the Oct4 (Oct3/4 index), Sox2 and Nanog regulatory network [2,3] is a significant advance, an integrated understanding is still lacking.

Some key approaches to understanding the molecular basis of pluripotency and early differentiation are the analysis of transcription factor binding site mapping [3], epigenetics studies (reviewed in [4]), as well as in-depth assessments of transcripts expressed in ES cells. Transcriptome surveys of ES cells by SAGE [5], MPSS [6,7], gene trapping [8] and EST sequencing [9,10] have been performed by several groups under the hypothesis that transcripts expressed specifically in ES cells are instrumental for maintaining pluripotency. Another transcript profiling method which has been used to interogate ES cell transcriptome and offers a marked improvement compared to those techniques is Gene Identification Signature (GIS) analysis [11].

GIS analysis is a SAGE modification which isolates tags of 18 base pairs (bp) from the 5'- and 3'-ends of a transcript and concatenates them to form Paired-End diTag (PET) structures. Whereas SAGE extracts a single tag per transcript, GIS analysis presents paired sequence from transcript start and end sites, marking the boundaries of transcriptional units (TUs) on the genome.

GIS analysis of mouse E14 ES cells generated 116,252 PET sequences. Among them were hundreds of novel, uncharacterized TUs readily apparent on comparison of PET boundaries with known-gene boundaries mapped to the genome. The novel TUs were separated into four categories: new transcriptional start and end sites of known genes, intergenic splicing connecting two adjacent genes as a single transcript, trans-splicing events, and totally novel transcripts.

Identification of novel transcripts from SAGE tags not matching known genes has been reported before [12,13], and heretofore unrecognized novel ES cell transcriptome components have been hypothesized to be involved in orchestrating the pluripotent state [5,8,9,14]. In one major recent study, ES-specific SAGE tags have been earmarked for 3' cDNA cloning and subsequent evaluation of the novel transcripts as potential regulators of the stemness phenotype [15].

We also hypothesized that some ES-derived novel TUs were expressed preferentially in undifferentiated ES cells, as they had not been identified previously with other transcriptome characterisation techniques, and hence that they may potentially be involved in maintaining pluripotency. Another example of this approach is the identification of Zfp206 as a transcription factor that controls pluripotency in ES cells [16]. Building up from large-scale EST and MPSS data which had indicated that the previously uncharacterized Zfp206 gene was differentially expressed between pluripotent and differentiated states, Wang et al. (2007) [16] found that Zfp206 regulates pluripotency and is actively involved in the Oct4 and Nanog regulatory loop.

To similarly accomplish discovery of novel pluripotency-associated transcripts, we focused on two groups of novel TUs inferred from GIS PET data: the fully novel transcripts and the intergenic splicing events. Since GIS analysis provided sequence from 5'- and 3'-ends of the transcripts, we physically validated and sequenced these TUs to reveal their intron-exon structures. We analysed their splice junctions, alternative isoforms, open reading frames (ORFs), and homologies to predict protein-coding potential. In an attempt to relate the TUs to ES-specific functions, we profiled their expression in tissue panels and differentiation timecourses, showing that the TUs were either ES-specific or downregulated upon differentiation. We outline five novel TUs to be further investigated for direct functional roles in ES cells.

## Results

### Initial computational analysis of novel transcriptional units in mouse embryonic stem cells

The initial dataset of 153 candidate unconventional PET-supported TUs consisted of 143 novel TUs not corresponding to known genes and 10 representing potential intergenic splicing. We eliminated TU candidates with multiple or ambiguous mappings, as described in Materials and Methods. Additionally, one novel and one intergenically spliced TU on the mm3 assembly matched known genes on mm6 and were not analysed further. Table 1 summarizes filtering results for novel and intergenically spliced candidate TUs with E14 ES PET support.

**Table 1:** Annotation-based filtering of candidate novel and intergenically spliced PET-based TUs.

Selection process	Initial dataset	Genomic span > 200 kb	Halftag/s within repeats	Mapping to multiple loci	Unmappable to the genome	No longer novel	Final PETs
Novel	143	48	48	26	10	1	10
Intergenic Splicing	10	-	-	-	3	1	6
**Total**	**153**	**48**	**48**	**26**	**13**	**2**	**16**

The numbers refer to PET counts in each named category corresponding to annotation-based filtering described in the text. The 'Mapping to multiple loci' and 'Unmappable to the genome' columns are the combined results of both In-Silico PCR and BLASTN.

16 TUs were chosen for experimental validation: 10 novel TUs with no known-gene support and 6 intergenically spliced TUs. Their corresponding PETs were visualized against the mm6 assembly to gauge the extent of mRNA and EST support (the number of individual publicly available GenBank mRNAs and ESTs, respectively, whose exons match one or both halves of a PET-transcriptome tag in the same transcription orientation as the tag itself) for these TUs. Eight TUs were supported at least partially by full-length cDNAs and/or ESTs, but the other eight lacked support in public transcriptome databases and were therefore completely novel (see Additional file 1 for the characterization of these 16 TUs).

### Expression validation, sequence analysis, and genomic properties of novel TUs in mouse embryonic stem cells

We assayed the 16 novel TUs in mouse ES cells by RTPCR. We obtained PCR products for 14 of the TUs. They were sequenced and BLAT-aligned to the genome.

Four TUs (TUs 3, 8, 12 and 15) yielded non-specific RTPCR products which did not map to the expected locus after sequencing. For all of those TUs, there had been no cDNA or EST evidence of nearby transcription along the genome. As a result, only the PET half-tag (5'tag and 3'tag [11]) sequences themselves were available for primer design. The short length and low complexity of these sequences likely resulted in suboptimal primers which amplified non-specific products.

Three other TUs (TUs 6, 9 and 10) gave specific products of sizes consistent with lack of splicing; two of these were also potentially problematic due to intronic same-strand localization relative to known genes. These seven TUs were therefore excluded. Of the other seven PCR-positive TUs, five yielded full-length sequences and became the focus of remaining analyses (see Additional file 2 for their full-length sequences).

#### TU4 (PET 1339): a novel PRY and SPRY domain-containing stem cell gene

**Figure 1:**
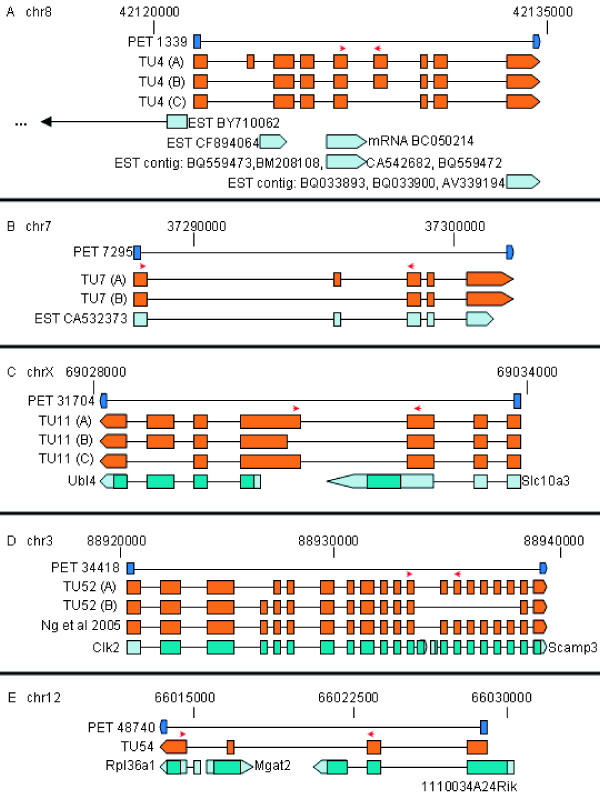
**Genomic structures of novel TUs**. A. TU4. B. TU7. C. TU11. D. TU52. E. TU54. Initial paired-end ditag: dark blue. Exons of validated full-length cDNA sequences: orange. Exons of previously known transcripts at each locus: teal (protein-coding sequence: shaded). 3' terminal exon arrows: direction of transcription. Quantitative real-time PCR primer locations: red arrows. All validated cDNA sequences can be found in Additional file 2.

Three isoforms of TU4 were recovered by full-length sequencing of RTPCR products. They were 1554, 1521 and 1397 bp long, named A [GenBank: EU599038], B [GenBank: EU599039] and C [GenBank: EU599040] respectively (Figure 1A). The longest isoform A had nine exons while isoforms B and C contained eight and seven, respectively. All splice junctions were canonical (GT-AG).

No spliced ESTs were present at this locus. Of the nine unspliced ESTs overlapping exonic sequences of TU4, one (CF894064) was from mouse ES cells and two (CA542682 and BM208108) were from trophoblast stem cells, as was the single full-length cDNA at the locus (BC050214). This cDNA- and EST-derived expression profile was consistent with expression of this TU in ES cells.

The three ESTs supporting the 3'-halftag of the original PET 1339 overlapped the last exon of TU4, while the other ESTs and the cDNA partially were internal to the TU. This implies that the diversity of TU4 full-length isoforms may be considerably greater than demonstrated here.

The longest isoform had an ORF of 424 amino acids (aa), from nucleotide (nt) 149 to nt 1423. This ORF contained the conserved domains PRY and SPRY, an evolutionary innovation associated with immunity [17]. It had homology to a predicted mouse protein similar to tripartite motif protein 39 and to a predicted rat protein similar to FLJ25801, which also contains PRY and SPRY domains.

Interestingly, TU4 shares a bidirectional promoter with BC050188, another uncharacterized protein-coding gene, through the EST BY710062. This EST constituted an alternative 5' end of BC050188 and was also of ES-cell origin. BC050188 itself was supported by many other ES-cell mRNAs and ESTs, and contained a partial PRY domain, though its ORF was only 79 aa long. However, its human putative ortholog, FLJ36180, had a 468-aa ORF with RING, PRY, and SPRY domains. The promoter sharing potential may imply a coregulatory and conserved relationship between these two tandemly arranged PRY-domain genes with ES-specific expression.

#### TU7 (PET 7295): a putative novel ES-specific transcription factor

TU7 had two isoforms, A [GenBank: EU599041] and B [GenBank: EU599042] (1492 and 1428 bp long), with five and four exons respectively. The splice junctions were canonical. Exonic same-strand overlap with known ESTs (Figure 1B), was observed. The structurally closest EST was CA532373, derived from a mouse ES cell line.

The longest ORF was 270 aa, coded by nt 107–919 of isoform B. The ORF contained partial KRAB and FOG zinc finger domains, homologous to a predicted protein similar to gonadotropin-inducible ovarian transcription factor 1. This suggests that TU7 may encode an ES-specific transcription factor.

#### TU11 (PET 31704): Slc10a3-Ubl4 intergenic splicing

TU11 connected the genes Slc10a3 and Ubl4. Taken separately, these two adjacent genes had numerous mRNAs and ESTs in the public databases, but no public transcripts connect the genes by intergenic splicing. We identified three isoforms of this TU from E14 ES cells, 1038, 1002 and 829 bp long, labeled A [GenBank: EU599043], B [GenBank: EU599044], and C [GenBank: EU599045] respectively (Figure 1C). All isoforms spliced the 5'UTR of Slc10a3 gene to a novel splice acceptor in the intergenic region and continued into the remaining sequence of the downstream Ubl4 gene. The two longer isoforms consisted of seven exons while the shortest had six, skipping one exon of Ubl4. All splice junctions, including the novel splice acceptors, were canonical, suggesting that this TU was biologically transcribed and not a result of cDNA library chimerism.

Only the two longer isoforms are likely to encode a protein; the 98-aa longest ORF of isoform C lacks any significant homology to Slc10a3, Ubl4, or any other proteins. The longer isoforms A and B both potentially encoded a 157-aa ORF. This ORF contained the conserved domain GDX_N in the first 61 aa and was homologous to Ubl4. BLASTP search of NCBI ORF Finder results did not detect any potential for chimeric protein formation, indicating that Slc10a3 contributed exclusively the 5'UTR to the Slc10a3-Ubl4 TU while the entire coding potential was contributed by Ubl4. Therefore, despite intergenic splicing, the transcript would not encode a novel protein. It is possible that regulatory sequences of Slc10a3, such as RNA-binding protein cognate sites in the 5'UTR, are functionally united with the Ubl4 ORF by this intergenic splicing and may result in Slc10a3 sequence-mediated effects on Ubl4 mRNA or protein levels.

#### TU52 (PET 34418): Clk2-Scamp3 intergenic splicing

**Figure 2:**
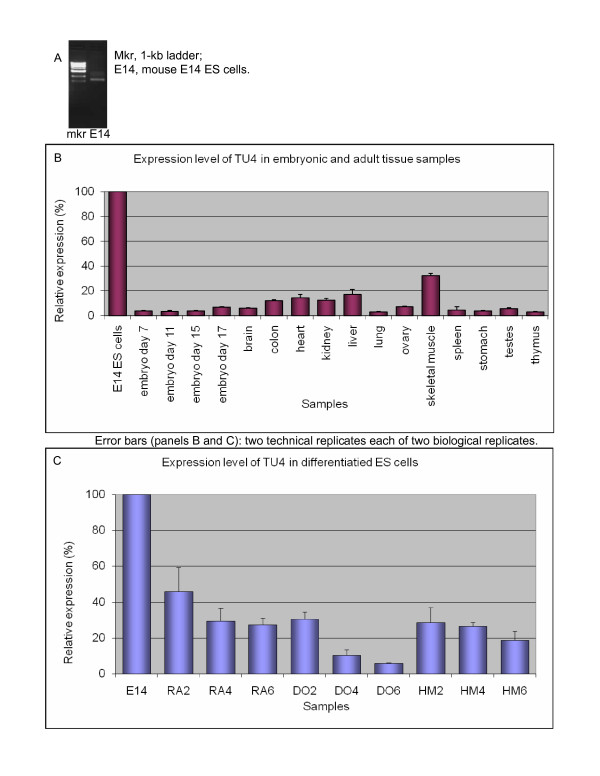
**Expression profile of TU4**. A. Qualitative RTPCR (samples testing negative are not shown). B. Quantitative RTPCR: whole-embryo and tissue panel. C. Quantitative RTPCR: retinoic acid (RA), dimethyl sulfoxide (DO), and hexamethylene bisacetamide (HM)-induced differentiation time courses of E14 ES (left to right: untreated; days 2, 4, 6).

TU52 connected the genes Clk2 and Scamp3. Each of the component genes had good mRNA and EST support individually but none of the public transcripts bridged the two genes into a single TU. In addition to the 2687-nt isoform of this TU [GenBank: EU599046] validated in [11] (Figure 2 of that publication), we identified two isoforms of 2599 and 1962 nt, labeled A [GenBank: EU599047] and B [GenBank: EU599048], respectively (Figure 1D).

Isoform A was similar to the 2687-nt published transcript [11] but skipped exon 4 of Clk2. It consisted of 19 exons, with all splice junctions being GT-AG. Isoform B had 14 exons and, likewise, all splice junctions were canonical. All three isoforms skipped the last exon of Clk2 and spliced into different Scamp3 splice acceptors, presumably as a consequence of transcriptional read-through. Isoform A and the published transcript spliced into the second exon of Scamp3 while isoform B spliced into the penultimate exon of Scamp3.

Isoform A had a 407-aa ORF (nt 703 to nt 1926) and, similarly to the published transcript, encoded a chimeric protein with components from both genes. The predicted protein contained the STKc (Serine/Threonine protein kinases, catalytic) domain from Clk2 and the SCAMP domain from Scamp3.

Clk2 may be involved in regulation of alternative splicing [18] and our result indicates that Clk2 itself may be alternatively spliced. Human SCAMP3 is known to undergo a specific tyrosine phosphorylation event, leading potentially to downregulation of the human EGF receptor [19]. The presence of the Clk2 kinase domain in the fused transcript might imply auto-regulation of the Scamp3 fragment of the chimeric protein by self-phosphorylation, a regulatory context distinct from conventional post-translational processing of Scamp3.

Isoform B had a longer ORF of 517 aa (nt 107 to nt 1660). However, this 517-aa longest ORF of the Isoform B mRNA only contained the STKc domain and was solely homologous to the Clk2 protein, because the Scamp3 protein sequence in this isoform was encoded in a different reading frame. Therefore, despite intergenic splicing, isoform B does not encode a chimeric protein.

#### TU54 (PET 48740): 1110034A24Rik-Rpl36al intergenic splicing

Intergenically spliced TU54 [GenBank: EU599049] connected the 1110034A24Rik and Rpl36al genes. The TU, 1147 bp long, has four exons with canonical splice junctions (Figure 1E). There were numerous cDNAs and ESTs supporting each gene separately, but none bridged the two. This TU overlapped in the reverse (cis-antisense) orientation the Mgat2 gene between 1110034A24Rik and Rpl36al.

The longest ORF of this TU was 166 aa (nt 278 to 778). It contained no conserved domains and was identical to the standalone 1110034A24Rik ORF. After the stop codon at nt 778 there was another 100-aa ORF identical to Rpl36al. Therefore, this intergenically spliced TU was potentially bicistronic, containing the complete ORFs of both component genes, but did not encode a fusion protein.

The cis-antisense overlap with Mgat2 was 58 nt, comprising the entire third exon of this TU which overlaps part of the coding sequence of Mgat2. Expression of the 1110034A24Rik-Rpl36al bicistronic TU may exert regulatory effect on Mgat through the cis-antisense pairing, as genes in other cis-antisense gene pairs can affect each other's expression [20].

We then investigated whether these new members of the ES cell transcriptome are regulated by known ES transcription factors. To accomplish this, we searched two datasets of experimentally supported transcription factor binding events for Oct4, Nanog, and Sox2 binding sites in the vicinity of the TUs: chromatin immunoprecipitation paired-end diTags (ChIP-PET) [3] and ChIP-sequencing by Solexa technology [H. H. Ng et al., unpublished]. In brief, all TUs had evidence of Oct4, Nanog, and/or Sox2 binding events within 100 kb of the TU in one or both types of ChIP datasets (See Additional file 3). Most notably, TU7 had Oct4 and Nanog binding sites 27.5 kb downstream of its 3' end, while TU52 had a Sox2 binding site 42.5 kb downstream of its 3' end supported by both Sox2 ChIP datasets. Only one binding event – a ChIP-seq Nanog binding site – was localized directly at the transcription start site, for TU4.

### Qualitative and quantitative expression profiling of novel TUs

Since the TUs were newly recognized components of the mouse ES cell transcriptome, the fact that they had never been detected before in any other cell type suggested that they might be preferentially expressed in ES cells. We thus hypothesized that they may be involved in the maintenance of pluripotency in these cells and accordingly, checked their expression in stem cells and a panel of embryonic and adult tissues, because higher expression in undifferentiated cells would be consistent with a function specific to such cells. An example of this pattern of gene expression is LIN28 which was found by SAGE to be expressed only in human ES cells [5] and showed downregulation upon ES cell differentiation. In fact, LIN28 has only been recently implicated in human somatic cell reprogramming back to the pluripotent state [21], indicating its importance in ES cells.

Conventional RTPCR and quantitative real-time PCR (QRTPCR) were used to profile expression of the novel TUs. The results, discussed further, showed that all five TUs were either ES-specific or were expressed most highly in ES cells despite being also transcribed elsewhere.

To further confirm the pluripotency association of the TUs, we also measured the changes in expression levels of the TUs in ES cells upon chemical induction of differentiation. ES cells were differentiated using retinoic acid (RA), dimethyl sulfoxide (DMSO), and hexamethylene bisacetamide (HMBA) treatments, in separate experiments. The three treatments served also to eliminate the possibility of chemical-specific expression responses.

To validate that the three chemical treatments reduced pluripotency marker levels and led the cells to differentiate, we tested expression levels of four principal pluripotency markers (Oct4, Sox2, Nanog and Klf4) by TaqMan QRTPCR. The results clearly indicated that all pluripotency marker mRNA levels decreased over time in all three treatment timecourses, proving that the treatments led to the expected differentiation outcomes (See Additional file 4).

QRTPCR was performed to determine the effect of differentiation on the RNA level of the novel TUs. We demonstrated that most of the TUs were consistently downregulated upon differentiation, regardless of the differentiation agents used.

#### TU4 (PET 1339): a novel stem cell gene with PRY and SPRY domains

Qualitative RTPCR on the multitissue panel suggested that TU4 had an ES-cell-specific expression pattern (Figure 2A and 2B). We did not detect expression of this TU outside of ES cells by RTPCR (data not shown). QRTPCR demonstrated that expression of this TU was low though not absent in non-ES samples. The second-highest expression level was in adult skeletal muscle, at 30% of the ES-cell level; in the few other tissues where the TU was expressed, its RNA level was less than 20% of the ES-cell level (Figure 2B). Consistent downregulation of this TU upon ES cell differentiation was evident in all three differentiation timecourses, although it was most pronounced with DMSO treatment (Figure 2C).

#### TU7 (PET 7295): a putative novel ES-specific transcription factor

**Figure 3:**
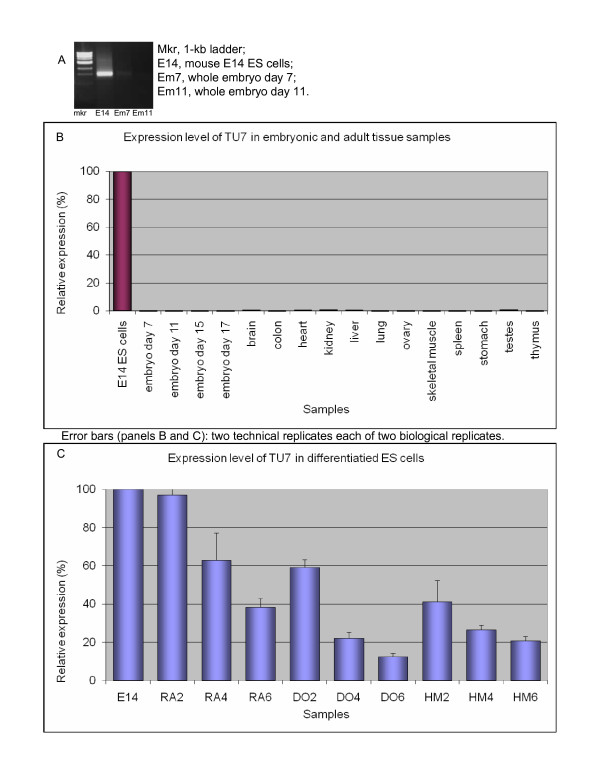
**Expression profile of TU7**. A. Qualitative RTPCR (samples testing negative are not shown). B. Quantitative RTPCR: whole-embryo and tissue panel. C. Quantitative RTPCR: retinoic acid (RA), dimethyl sulfoxide (DO), and hexamethylene bisacetamide (HM)-induced differentiation time courses of E14 ES (left to right: untreated; days 2, 4, 6).

This novel TU showed a similar expression pattern to TU4. Qualitative RTPCR indicated that besides expression in ES cells this TU had barely-detectable expression in the embryo at days seven and eleven (Figure 3A). QRTPCR confirmed the ES specificity of TU7 (Figure 3B).

The expression level of TU7, similarly to TU4, showed a decreasing trend upon ES cell differentiation (Figure 3C). This gradual effect was observed in all three treatments but the decrease was not as rapid as that of TU4. A significant reduction in TU7 expression was observed after four days of RA treatment whereas for TU4 the effect was observed as early as day two. In the other differentiation treatments, the effect was similar between the two genes, with DMSO treatment yielding the strongest response. This expression profile argues in favor of potential relevance of TU7 to ES cell pluripotency.

#### TU11 (PET 31704): Slc10a3-Ubl4 intergenic splicing

**Figure 4:**
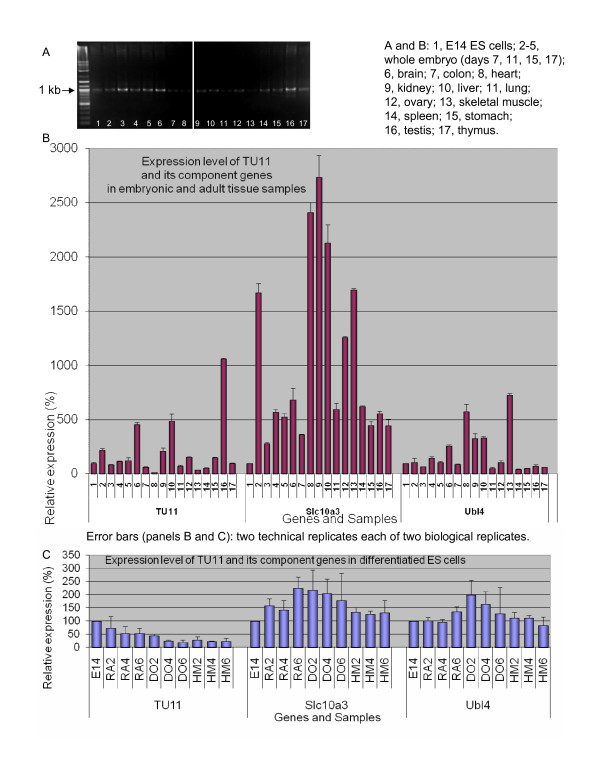
**Expression profiles of TU11, an intergenically spliced product of the Slc10a3-Ubl4 locus, and its component genes**. A. Qualitative RTPCR. B. Quantitative RTPCR: whole-embryo and tissue panel. C. Quantitative RTPCR: retinoic acid (RA), dimethyl sulfoxide (DO), and hexamethylene bisacetamide (HM)-induced differentiation time courses of E14 ES (left to right: untreated; days 2, 4, 6).

Qualitative RTPCR suggested that the intergenically spliced Slc10a3-Ubl4 TU had a close-to-ubiquitous expression profile (Figure 4A), although it nevertheless was preferentially expressed in embryonic rather than adult tissues. One adult sample with higher expression of Slc10a3-Ubl4 than ES cells was testes (Figure 4B). This is intriguing because adult testes reportedly contain multipotent cells capable of forming derivatives of the three embryonic layers [22].

QRTPCR showed that this bicistronic TU was downregulated upon differentiation (Figure 4C). DMSO and HMBA differentiation timecourses resulted in a more rapid downregulation of Slc10a3-Ubl4, giving it an expression profile recapitulating that of TU7.

To verify that this intergenically spliced TU was involved in pluripotency but its component genes were not, we individually measured expression levels of the component genes, Slc10a3 and Ubl4. A correlation between the expression level of the bicistronic TU and expression levels of its component genes would suggest that Slc10a3-Ubl4 was not an independently regulated TU.

However, the expression level of this TU was markedly different from those of the component genes, both in the multitissue panel and in all three differentiation timecourses (Figure 4B and 4C respectively). In the multitissue panel Slc10a3 was highly upregulated in adult tissues, especially in heart, kidney, and liver, while Ubl4 expression was high in the heart and skeletal muscle relative to E14 ES cells. In the differentiation timecourses, the component genes did not show any down regulation effect upon induction of differentiation. In fact, they were upregulated, especially upon DMSO treatment. This result showed that although this bicistronic TU contained the 5' UTR of Slc10a3, its regulation did not simply reflect the expression profile of that gene. We conclude that Slc10a3-Ubl4 is a genuine novel transcript regulated independently of its component genes. Furthermore, we propose that this locus may undergo expression regulation by a combination of alternative splicing and preferential transcription initiation at the downstream Ubl4 promoter, such that intergenic splicing is favored in ES cells while independent transcription of the component genes occurs preferentially in differentiated tissues.

#### TU52 (PET 34418): Clk2-Scamp3 intergenic splicing

**Figure 5:**
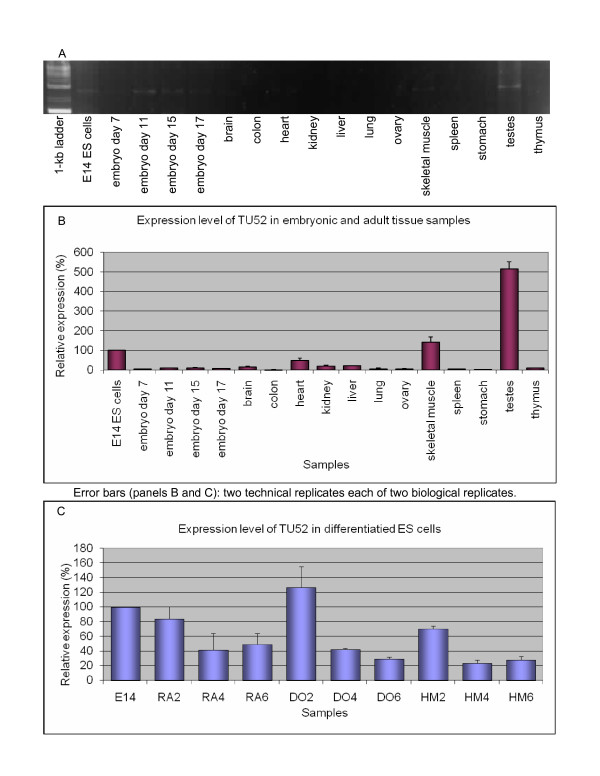
**Expression profile of TU52, an intergenically spliced product of the Clk2-Scamp3 locus**. A. Qualitative RTPCR. B. Quantitative RTPCR: whole-embryo and tissue panel. C. Quantitative RTPCR: retinoic acid (RA), dimethyl sulfoxide (DO), and hexamethylene bisacetamide (HM)-induced differentiation time courses of E14 ES (left to right: untreated; days 2, 4, 6).

Based on qualitative RTPCR, TU52 appeared largely ES-specific, with some embryonic expression (Figure 5A). The only adult tissue samples that showed a visible band were skeletal muscle and testes. QRTPCR showed that expression level in testes was approximately fivefold greater than the ES-cell level (Figure 5B).

Upon ES cell differentiation, the Clk2-Scamp3 TU showed significant downregulation (Figure 5C). In RA- and HMBA-treated cells the gene showed a similar expression profile with a maximum decrease to 20% of the ES-cell level after four days of treatment. In the DMSO timecourse this effect was less pronounced.

#### TU54 (PET 48740): 1110034A24Rik-Rpl36al intergenic splicing

**Figure 6:**
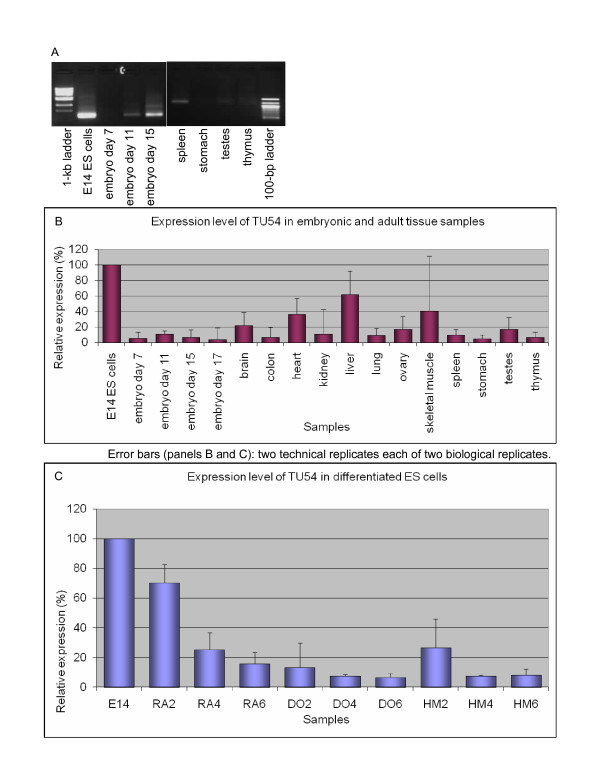
**Expression profile of TU54, an intergenically spliced product of the 1110034A24Rik-Rpl36al locus**. A. Qualitative RTPCR (samples testing negative: not shown). B. Quantitative RTPCR: whole-embryo and tissue panel. C. Quantitative RTPCR: retinoic acid (RA), dimethyl sulfoxide (DO), and hexamethylene bisacetamide (HM)-induced differentiation time courses of E14ES (left to right: untreated; days 2, 4, 6).

Qualitative RTPCR of the intergenically spliced 1110034A24Rik-Rpl36al TU showed that it was not ES cell-specific, as multiple embryonic and adult samples showed detectable expression (Figure 6A). However, QRTPCR indicated that the highest expression level was still in ES cells (Figure 6B).

Accordingly, RA, DMSO and HMBA treatments all showed that expression of 1110034A24Rik-Rpl36al decreased as the cells differentiated (Figure 6C). This decrease was significant in all of the timecourses tested with up to 80% reduction in mRNA expression in RA-treated cells at day six and up to 90% decrease in both DMSO- and HMBA-treated cells.

### RNAi-based functional analysis of novel TUs

**Figure 7:**
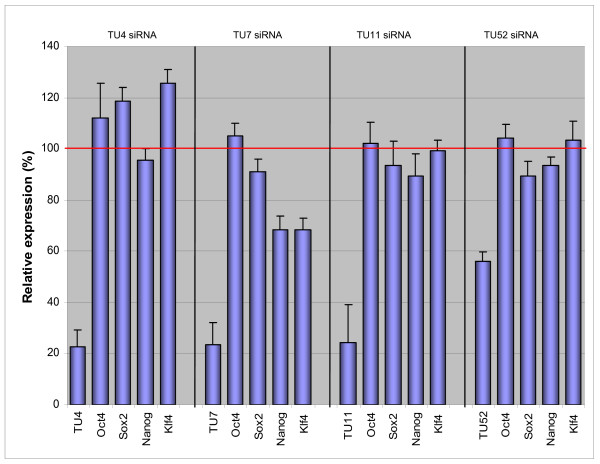
**Expression levels of pluripotency markers upon siRNA knockdown of the novel TUs**. Nanog and Klf4 expression was reduced to 70% upon knockdown of TU7 but no down regulation of the other pluripotency markers was observed. There was no down regulation of Oct4, Sox2, Nanog and Klf4 upon knockdown of the other novel TUs. Expression level was measured relative to Non-Targeting siRNA control (100%). Error bars indicate S.E.M. from two biological replicates.

We designed 4-siRNA combinations targeting TU4 and TU7 and individual siRNAs uniquely targeting the intergenically spliced TU11, TU52, and TU54 transcripts (see Methods for design notes). We were unable to achieve satisfactory TU54 knockdown (data not shown). We accomplished knockdowns of the other four TUs to 25% – 55% of control (non-targeting siRNA transfection) mRNA levels (Figure 7).

We hypothesized that, if the novel TUs were relevant to maintaining ES cell pluripotency instead of merely associated with pluripotency, then depletion of RNA levels of the TUs could result in depletion of mRNA levels of some, or all, of the four principal pluripotency-driving transcription factors (Oct4, Sox2, Klf4, and Nanog). Although both Nanog and Klf4 expression levels were reduced to 70% of reference upon knockdown of TU7, no downregulation of the Oct4 or Sox2 was observed. There was no downregulation of Oct4, Sox2, Klf4, or Nanog upon knock down of the other novel TUs (Figure 7).

We also reasoned that, if the TUs were indeed required for maintaining the ES state, then RNAi suppression of the TU RNA levels could lead to upregulation of well-known ectodermal, endodermal, and mesodermal lineage markers. We therefore tested, by QRTPCR with commercially available primer sets and fluorescent probes (TaqMan, Applied Biosystems), the mRNA levels of ectodermal (Fgf5, Nestin, Sox1, Sox4, Pax6, Pax7, Rest), endodermal (Sox17, Gata4, Gata6, Foxa2, Afp), and mesodermal (Gata2, Nkx2.5, T-brachyury, MyoD, Hand1, and Bmp4) differentiation markers after independent TU4, TU7, TU11, and TU52 RNAi transfections.

Differentiation marker levels were unaffected by TU4, TU11, and TU52 RNAi. Upregulation of Fgf5 (but not any other ectodermal markers) and T-brachyury (but not any other mesodermal markers), to approximately 150% of reference levels each, was observed at day 3 and day 5 post-TU7 RNAi (data not shown). Nanog, Klf4, Fgf5, and T-brachyury were reproducibly affected by TU7 perturbation, making a direct function of TU7 in pluripotency maintenance possible in principle. However, the lack of generalized effects on pluripotency transcription factors and differentiation marker classes suggests that even TU7 may not be an essential pluripotency regulator.

## Discussion

### Validation rate of novel TUs from a PET-transcriptome library

The five novel TUs whose transcription and full-length sequence we validated represent only a small subset (3.3%) of candidate novel TUs revealed by PET-based mES transcriptome sampling. We ascribe this small fraction to our selection criteria implemented in selecting TUs to be validated. The criteria were designed to maximize the chance of experimental validation but do not imply that all remaining TUs are artefactual.

To sample a larger number of TUs for future physical validation, selection criteria can be relaxed. For example, the 200 kb cutoff value for maximum genomic span can be increased since some genes are an order of magnitude longer (for example, Dmd [23]). Two putative bicistronic TUs which were excluded from the original dataset would have been detected if the cutoff had been increased to 1 Mb.

Among TUs selected for physical validation, only five yielded specific products. One reason for the low efficiency is likely the limited PET sequence available for primer selection, the crucial point of the validation process. It is possible that the primers designed from the PET sequence were suboptimal and unable to bind to the correct transcript resulting in both false negative and false positive PCR results. The presence of ESTs and flcDNAs in the locus clearly aids validations as it increases the amount of known exonic sequence space putatively belonging to the PET-supported TU; utilizing this space benefits primer design.

Ng et al. (2005) verified a large proportion of PET-supported mES transcripts (94.4%). However, they used their own GIS flcDNA library as the template source. Thus, some of their confirmed TUs were likely due either to experimental artefacts or to extremely rare transcripts which were clonally propagated in that library, but might not be present at detectable levels in normal E14 cells. We used RNA from E14 cells for our analysis. Furthermore, the high success rate obtained by Ng et al. was for a combination of novel and known genes; the validation rate for novel TUs alone was not explicitly specified. These factors might account for our lower validation rate.

### Intergenic Splicing

Three of our five novel TUs join two adjacent genes by forming a single transcript containing exonic sequences of both genes. Despite sporadic reports of intergenic splicing (reviewed in [24,25]), its biological significance is largely unknown. Several models speculate on how such transcriptional gene fusion may have a physiological role. First, the fusion may be a byproduct of transcriptional read-through, whereby the transcriptional machinery does not stop at the transcription stop site of the first gene but "reads-through" to the 3' end of the next gene on the same strand instead. These can be rare unregulated stochastic events with no functional significance. However, we showed that our transcripts were not present just in ES cells; they were frequently detectable in several, though not all, cell and tissue types, but never ubiquitous, and the expression levels differed reproducibly between samples. Consequently, the intergenic splicing events we observed are unlikely to be random.

Second, chimeric TUs – those with fusion ORFs in addition to intergenic splicing – can form novel and truly functional proteins. The fusion can create a new bifunctional protein, as is the case in the TWEAK-APRIL fusion [26], or change the properties of one of the participating proteins. An example of the latter case is the Kua-UBE2V1 fusion [27] where the fused protein is cytoplasmic while the original UBE2V1 is nuclear. Only one of our three intergenically spliced TUs has a putatively chimeric fusion ORF, however. Only 25% of intergenically spliced genes detected in a recent study encode chimeric proteins [24].

A less clear functional impact of intergenic splicing centers on expression regulation. A theoretical example is the bicistronic TU11 which contained the 5' UTR of the upstream Slc10a3 gene and the entire coding sequence of the downstream Ubl4 gene. Since TU11 contained the 5'UTR of Slc10a3 it could be hypothesized that the bicistronic transcript would be co-regulated together with the upstream gene. However, analysis of their expression level showed that TU11 was regulated independently from its two component genes. There might be other consequences of the hybrid "5'UTR of gene 1 – ORF of gene 2" composition of this TU, for example potential for regulation of Ubl4 by RNA-binding proteins which only recognize specific sites in the 5'UTR of Slc10a3.

Despite the scale of the intergenic splicing datasets reported in [24] and [25], neither study identified intergenic splicing at the three loci corresponding to our TUs. In fact, even a recent high-throughput effort to catalog novel components of the mouse ES transcriptome [8] did not yield any evidence of intergenic splicing at our three loci, although gene trap tags corresponding individually to Ubl4, Clk2, Scamp3, and Rpl36al were isolated in that project. This shows that diversity and genomewide incidence of this phenomenon exceed current estimates, and emphasizes the need for targeted validation of candidates from specific libraries, such as our mES transcriptome library, to gain insight into the diversity of intergenic splicing in transcriptomes previously not subjected to deep sampling.

### Relevance to pluripotency of ES cells

In addition to reporting novel TUs in mouse ES cells, we provide preliminary evidence for their functional significance. Since the novel TUs were initially found in undifferentiated ES cells, we hypothesized that they were involved in ES cell pluripotency. Our findings initially supported this hypothesis since almost all of the TUs we reported here were preferentially expressed in ES cells compared to adult tissues, and all TUs had nearby Oct4, Sox2, and Nanog binding sites confirmed by two independent lines of ChIP evidence. This suggests an importance of these TUs in ES, rather than differentiated, cells, although it does not preclude functional roles in other contexts.

More direct support for our hypothesis is provided by the downregulation of these TUs upon ES cell differentiation. The effect was common to all three differentiation treatments tested, indicating that it was not a chemical-specific response, but rather a reproducible outcome of differentiation. Combined with the EST-derived expression profiles and multi tissue expression analysis, this result further implicates the five TUs in maintaining ES cell pluripotency.

Loss-of-function experiments for four of these novel TUs did not lead to cell differentiation as can be seen from the levels of pluripotency markers Oct4, Sox2, Nanog and Klf4 upon siRNA transfections. However, TU7 was intriguing because its knockdown led to approximately 30% concurrent reduction in Klf4 and Nanog levels, as well as to 1.5-fold upregulation of Fgf5 and T-brachyury. It has been known that T-brachyury is able to upregulate Nanog, which in turn suppresses T-brachyury's own expression in an indirect, Smad-facilitated negative feedback loop in early mesodermal progenitors [28,29]. If an identical T-brachyury/Nanog relationship exists in undifferentiated ES cells or in an early mesodermal progenitor subpopulation thereof, then it is possible that TU7 may be a contributor in maintaining Nanog levels in undifferentiated ES cells so that when its expression is knocked down Nanog level is no longer maintained and the suppression of T-brachyury is lost.

Summarily however, these five TUs alone, without unknown cofactors, are not necessary or sufficient for maintaining pluripotency. Overexpression of the TUs, in transient or stable transfectants, could be attempted as an additional functional test in order to determine whether higher levels of the TU RNAs enable ES cells to resist chemical differentiation drivers. While an in-silico or computational subtractive-hybridization comparison with a non-ES transcriptome would be appropriate, there was no non-ES PET transcriptome dataset generated by Ng et al. [11], precluding direct quantitative comparison of these novel TUs' expression in ES vs. non-ES states.

## Conclusion

Our observations summarily demonstrate that all five TUs we analyzed represent genuine novel transcripts potentially important in maintaining pluripotency. To elucidate the biological importance of these novel TUs, RNAi knock-downs were performed, which indicated that RNA level ablation of TU7 leads to a modicum of reproducible expression profile changes associated with the three principal cell differentiation fates while such ablation of the other four TUs failed to change expression phenotypes. Nevertheless, our results establish a foundation for functional analysis of a small number of novel TUs filtered from a large set of candidates, and show that the GIS mES PET-transcriptome library is a good resource for identification of novel TUs highly expressed in, and specific to, ES cells.

## Methods

### GIS dataset

TUs not corresponding to known genes were obtained by screening the transcriptome data from [11] for PET clusters which were > 10 kilobases (kb) away from known genes at one or both ends. TUs with putative intergenic splicing were obtained from Supplementary Table 3 of that publication. PET index numbers, paired halftag sequences, and PET alignments to the mm3 genome assembly were retrieved from the sme003_63467Apr22b dataset at the T2G public website [30] (See Additional file 1 for the detailed PET sequences used in this analysis).

### Computational analysis

#### Genomic alignments and repeat content of PET sequences

BLAT [31,32] was used to align PET sequences and the full-length TU sequences we obtained to the mouse mm6 genome assembly, the latest available as of the inception of the project. The RepeatMasker track of the UCSC Browser was used to determine whether each PET halftag localized to repetitive sequence. 48 PETs were removed because they overlapped repetitive elements.

The halftag sequences of each PET were used as UCSC in-silico PCR [33] primers on the mm6 assembly. Default parameters were used, with a 200-kb maximum product size to reduce potential artifacts; 10 PETs were removed due to this threshold. Three PETs that were shown to ambiguously map to multiple genomic loci and six PETs that did not return any result, indicating that their halftags were not present in tandem on the mm6 genome assembly, were subsequently eliminated.

#### Specificity check of non-repetitive PETs with unique mappings

NCBI BLASTN with the "Search for short, nearly exact matches" option [34] was run separately for each halftag of each PET, using default parameters and limited to the *Mus musculus *subbase of the NR database. The goal of this analysis was to eliminate halftag sequences which, despite unique in-silico PCR mappings, could compromise RTPCR primer design. This eliminated 23 PETs which mapped to multiple loci, and seven PETs that were unmappable to the genome or mapped to a different chromosome than initially predicted.

#### Primer design

For each halftag of each PET, the end internal to the genomic span of the underlying transcript (not the end corresponding to the transcript boundary) was lengthened by at least three to five nt (extending toward the other halftag of the PET) to generate 21 – 23 nt candidate primers. The primer design criteria were: Tm of 50 to 65°C, GC content of 45 to 60% with no poly-G or poly-C mononucleotide runs over 3 nt long, a GC clamp at the 3' end, no palindromes, dimers nor hairpin loops, and Tm difference between the forward and reverse primers of less than 10°C. If a primer was unacceptable, new candidate primers were obtained by sliding further into the genomic span of the PET. For halftags supported by exonic overlap with cDNAs or ESTs on the same strand, we assumed that the cDNAs/ESTs and the PET represented the same TU, and incorporated the extent of cDNA/EST exons for higher-confidence primer design instead of limiting primer selection to several bases inwards from the PET boundaries (see Additional file 5 for primer sequences used in this analysis).

#### Coding capacity evaluation

The ORF Finder [35] at the NCBI website was used to identify all potential positive-strand ORFs in the full-length TU sequences which we obtained from RTPCR products. The longest positive-strand ORF was selected as the most probable ORF. BLASTP search was performed to find its putative conserved domains and homologies to known protein sequences.

### Cell culture

E14 mouse ES cells (ATCC) were cultured, feeder-free, in gelatin-coated culture dishes containing Dulbecco's modified Eagle's medium (GIBCO), supplemented with 15% ES cell-qualified fetal bovine serum (GIBCO), 2 mM L-glutamine, 0.1 mM MEM non-essential amino acids, 0.055 mM beta-mercaptoethanol (GIBCO) and 1000 units/ml of LIF (Chemicon). Cells were maintained at 37°C with 5% CO2. For differentiation treatments, cells were cultured as above but without LIF, and supplemented with one of the differentiation agents. The three agents were: 0.1 μM retinoic acid (RA), 1% dimethylsulfoxide (DMSO) or 3 mM hexamethylene bisacetamide (HMBA).

### RNA extraction and RTPCR

Total RNA was extracted using TRIzol reagent (Invitrogen) and purified with the RNAeasy Mini Kit (QIAGEN). Mouse multi-tissue RNA was purchased from BD Biosciences Clontech (see Additional file 6 for the catalog numbers). Reverse transcription of 1 μg total RNA was performed using oligo(dT) priming with the SuperScript II Kit (Invitrogen). PCR was performed using Platinum Taq (Invitrogen) with 5% DMSO and the following thermocycler profile: initial denaturation step at 95°C for 10 minutes; 95°C for 30 s followed by primer annealing temperature for 30 s and elongation at 72°C for 1 minute for 40 cycles; final extension step at 72°C for 10 minutes. PCR products were analyzed on 1.5% agarose gels stained with ethidium bromide. Whenever the first round of PCR did not yield any product, a second round of PCR using either the same primers or nested primers as listed in Additional file 5 was performed on template generated by the first PCR reaction because failure to detect a band following the first reaction could have been due to the low expression level of the original transcript.

### PCR purification, gel extraction, cloning and sequencing

Depending on the gel electrophoresis result, the PCR product was either purified using the QIAquick PCR Purification Kit (QIAGEN) or, if multiple PCR products were present on the gel, the band of interest was excised and purified using QIAquick Gel Extraction Kit (QIAGEN). The TOPO TA Cloning Kit (Invitrogen) was used to clone the purified products into TOP10 cells. Plasmid DNA was isolated using QIAprep Miniprep Kit (QIAGEN) prior to sequencing using M13 Forward (-20) and Reverse primers.

### Quantitative Real-Time PCR

cDNA synthesis of 2 μg total RNA was performed using High Capacity cDNA Archive Kit (Applied Biosystems) followed by ten-fold dilution of the product. ABI Prism 7900 Sequence Detection System was used for the QRTPCR analysis. TaqMan Universal PCR Master Mix (Applied Biosystems) with commercially available prefabricated TaqMan probes (for pluripotency transcription factors, differentiation markers) and custom-designed TaqMan probes (for TU11), or SYBR Green Master Mix (Applied Biosystems) with 5 μM of forward and reverse primers (for the other transcript sequences) were used as the reagents in a total volume of 10 μl per sample in a 384-well standard plate. Results were analysed using the Relative Quantification (ΔΔCt) method using β-Actin as reference standard. Please see Additional file 7 for the catalog numbers of TaqMan probes used in the QRTPCR reactions.

### Small Interfering RNA (siRNA) Experiments

Custom siRNAs (Dharmacon) were designed against the novel TUs using siDESIGN Center [36]. For the intergenic splicing TUs the siRNAs were designed against the unique exon or against the unique exon-exon boundaries in TU11, TU52, and TU54 respectively so as not to affect the expression of their component genes. Dharmacon siCONTROL Non-Targeting siRNAs were used as the negative control. Mouse E14 ES cells were transfected using DharmaFECT 2 Reagents (Dharmacon) according to the manufacturer's instruction at a density of 1.5 × 10^5 ^cells per well in a 12-well plate. Retransfections were performed at 48-h intervals and the cells were harvested for analysis on day 3 (for QRTPCR of differentiation markers) and day 5 (for QRTPCR of pluripotency transcription factors). Please see Additional file 8 for the custom siRNA sequences used in the experiment.

## Abbreviations

ES (cell): embryonic stem (cell). TU: transcriptional unit. ORF: open reading frame. UTR: untranslated region. EST: expressed sequence tag. cDNA: complementary DNA sequence of a transcript. MPSS: massively parallel signature sequencing. SAGE: serial analysis of gene expression. RNAi/siRNA: RNA interference/small interfering RNA. GIS (analysis): Gene Identification Signature analysis. PET: paired-end ditag. RTPCR: reverse transcriptase – polymerase chain reaction. QRTPCR: quantitative real-time PCR.

## Authors' contributions

LL, GK, and LWS designed the project. LL and GK conducted all in-silico sequence analyses. GK and KYW performed the experiments. LWS provided critical guidance. All authors read and approved the final manuscript.

## Supplementary Material

Additional file 1 Detailed PET sequences used in this analysis and the genomic properties of the 16 novel and putative intergenically spliced TUs selected for experimental validation from the GIS PET-transcriptome dataset. Click here for file

Additional file 2 Full-length sequences of all splice isoforms of all the 5 TUs studied and the intergenically spliced transcript found in [11]. Click here for file

Additional file 3 Oct4, Nanog, and Sox2 binding sites in the 100 kb vicinity of the TUs identified from chromatin immunoprecipitation paired-end diTags (ChIP-PET) [3] and ChIP-seq [H. H. Ng et al., unpublished]. Click here for file

Additional file 4 Expression levels of the pluripotency markers Oct4, Sox2, Nanog, and Klf4 upon RA-, DMSO-, and HMBA-induced differentiation. Click here for file

Additional file 5 Primer sequences used to characterize the TUs identified in this study. Click here for file

Additional file 6 Catalog numbers of mouse total RNA (BD Biosciences Clontech) used in the multi-tissue panel expression analysis. Click here for file

Additional file 7 Catalog numbers of TaqMan probes (Applied Biosystems) used in the QRTPCR. Click here for file

Additional file 8 Sequences of the custom siRNA (Dharmacon) designed for the TUs. Click here for file
