# Supplementary material for: Detailed characterization of the mouse embryonic stem cell transcriptome reveals novel genes and intergenic splicing associated with pluripotency
Source: BMC Genomics. 2008 Apr 9;9:155. doi: 10.1186/1471-2164-9-155 (PMC2375908; doi:10.1186/1471-2164-9-155)
Supplement: Additional file 2 — Full-length sequences of all splice isoforms of all the 5 TUs studied and the intergenically spliced transcript found in [11]. [file 1471-2164-9-155-S2.pdf]

Full-length sequences of all splice isoforms of all the 5 TUs studied and the intergenically spliced transcript found in [11]

>TU4_isoform_A
GTACTGAAGTCTCTGGTCTCCACTGGCTGTGGCTTTCCCTGAAGGAAATCCTTAAGATATTTCTGGAAAGGAAGGGCGCTGGGATACCA
AGGAAGAAGTCCACATCGGAAACAAGCAGTCGAGTGTTCATCTGGTGCCACTGACTAGAATGTCGCAAGCGCCCAAGCCCCAGTTACCT
GGCAACACAGAGGATCCAAACTGCGGGACACACCCGGAGCCATCGCAGCTCTTCAGTGATGATGACCAAGTCATAGTTTGCAGCAAATG
TTTCCAGTCTCCAGAGCACAAGCAGCATGGGGCATACGGTATCCAAGAAGCTGCTGAGTATTACAGGATGTTACTCCAGGAAACCCTGG
TTACATTGAGGGAGAAGCGTGATGCAGCTACGAGCTTACTGGCCGAGGAGCGAGAGAGAATGGTGACGATTCAGGAAGAAGAGCAGAGG
TTTAAAGAGATGATCGAGGCTGAGTATAAGATGCGATTCCAGTTACTGACTGAAGAGGAGGAACTGGCAGCCTACAAAGTTGACCTGAA
CTTAAATGGACCTAATCCAGACCAGCTGATGAAGTGTGGCACTGAGCTCATGCAGAAGTCCCAAGAAATGCTACAGAGACTTAGTCACC
TGGGGAGAGAGAACATGGAAAAACTGAAGGCGAGTGAAGTCCGGCTTGGTGAGCACCTGCGCAGCCTCCAGACAATGATCACGGACCTC
GAGAAGAACTGTGGGGAATCTGCCGTTGCACTGCTCCAGAATGCAAAGTGCTATTTAAAAAGGAATGAGTCTATACTGCTTCAGAGCCT
AGAGCCAGCTCAGATAACAGACCTGAGTTCCTGCCAGATAATCGAAACGAGCCGTGTTCTACTGAGGCTCCAAAGACATATTACTTTGG
ACCCAGGCACAGCTCACCCCTCCCTAGTCTTATCCGAAGACCTGAGAGGTGTGGGATTCCGAGAGACACCAAAGACTGGGCCTGGCACT
ACAAGGAGATTGGATTTCGGCGCTTCTGTGTTGGGTGCAGAGAGCTTCACCACGGGGAGGCATTATTGGGAGGTGGCTGTGGGACAGGC
AACCCAATGGCAGGTGGGCATATGTGACTGTACAGAGAGAAAGGGCAACATTCCTGGGGCTTCTGGAGATAAAGTCTTGCTCATGGGGT
CCATGATGGGGACCGATTGTACCCTCTGGGTCTTTCCCCCTTTAAGAAAGGTCTGCCTGAGAAACCAAATGTACAAAGTTGGAGTCTTC
CTAGACTGTGAATGTGGGCAAGTATCCTTCTACAACGTGACAGAGCAGTCCCTCATTTACAGTTTCTCTGACCTTACCTTCCGAGGAGC
CGTTAAACCAATATTTTCTCTTTGTATTCCAAATGGAGGCATGAGTTCAGACTCTCTCACTGTCTGTCTTTCTCAGACTCATCCCTGAA
ATGCTGCTGATAGCTCTTTATCTTCCTGTGTGTGGAACCCAAGATCAAAAGAGACACAAAAATAAGATCCAGACTTTGTGAACATCACT
TATGTAATTAATCTAATATAATCCAATAAAGCCATTAGACT

>TU4_isoform_B
GTACTGAAGTCTCTGGTCTCCACTGGCTGTGGCTTTCCCTGAAGGAAATCCTTAAGATATTTCTGGAAAGGAAGGGAAGAAGTCCACAT
CGGAAACAAGCAGTCGAGTGTTCATCTGGTGCCACTGACTAGAATGTCGCAAGCGCCCAAGCCCCAGTTACCTGGCAACACAGAGGATC
CAAACTGCGGGACACACCCGGAGCCATCGCAGCTCTTCAGTGATGATGACCAAGTCATAGTTTGCAGCAAATGTTTCCAGTCTCCAGAG
CACAAGCAGCATGGGGCATACGGTATCCAAGAAGCTGCTGAGTATTACAGGATGTTACTCCAGGAAACCCTGGTTACATTGAGGGAGAA
GCGTGATGCAGCTACGAGCTTACTGGCCGAGGAGCGAGAGAGAATGGTGACGATTCAGGGAGAAGAGCAGAGGTTTAAAGAGATGATCG
AGGCTGAGTATAAGATGCGATTCTAGTTACTGACTGAAGAGGAGGAACTGGCAGCCTACAAAGTTGACCTGAACTTAAATGGACCTAAT
CCAGACTAGCTGATGAAGTGTGGCACTGAGCTCATGCAGAAGTCCCAAGAAATGCTACAGAGACTTAGTCACCTGGGGAGAGAGAACAT
GGAAAAACTGAAGGCGAGTGAAGTCCGGCTTGGTGAGCACCTGCGCAGCCTCCAGACAATGATCACGGACCTCGAGAAGAACTGTGGGG
AATCTGCCGTTGCACTGCTCCAGAATGCAGAGTGCTATTTAAAAAGGAATGAGTCTATACTGCTTCAGAGCCTAGAGCCAGCTCAGATA
ACAGACCTGAGTTCCTGCCAGATAATCGAAACGAGCCGTGTTCTACTGAGGCTCCAAAGACATATTACTTTGGACCCAGGCACAGCTCA
CCCCTCCCTAGTCTTATCCGAAGACCTGAGAAGTGTGGGATTCCGAGAGACACCAAAGACTGGGCCTGGCACTACAAGGAGATTGGATT
TCGGCGCTTCTGTGTTGGGTGCAGAGAGCTTCACCACGGGGAGGCATTATTGGGAGGTGGCTGTGGGACAGGCAACCCAATGGCAGGTG
GGCATATGTGACTGTACAGAGAGAAAGGGCAACATTCCTGGGGCTTCTGGAGATAAAGTCTTGCTCATGGGGTCCATGATGGGGACCGA
TTGTACCCTCTGGGTCTTTCCCCCTTTAAGAAAGGTCTGCCTGAGAAACCAAATGTACAAAGTTGGAGTCTTCCTAGACTGTGAATGTG
GGCAAGTATCCTTCTACAACGTGACAGAGCAGTCCCTCATTTACAGTTTCTCTGACCTTACCTTCCGAGGAGCCGTTAAACCAATATTT
TCTCTTCGTATTCCAAATGGAGGCATGAGTTCAGACTCTCTCACTGTCTGTCTTTCTCAGACTCATCCCTGAAATGCTGCTGATAGCTC
TTTATCTTCCTGTGTGTGGAACCCAAGATCAAAAGAGACACAAAAATAAGATCCAGACTTTGTGAACATCACTTATGTAATTAATCTAA
TATAATCC

>TU4_isoform_C
GTACTGAAGTCTCTGGTCTCCACTGGCTGTGGCTTTCCCTGAAGGAAATCCTTAAGATATTTCTGGGAAGGAAGGGAAGAAGTCCACAT
CGGAAACAAGCAGTCGAGTGTTCATCTGGTGCCACTGACTAGAATGTCGCAAGCGCCCAAGCCCCAGTTACCTGGCAACACAGAGGATC
CAAACTGCGGGGCACACCCGGAGCCATCGCAGCTCTTCAGTGATGATGACCAAGTCATAGTTTGCAGCAAATGTTTCCAGTCTCCAGAG
CACAAGCAGCATGGGGCATACGGTATCCAAGAAGCTGCTGAGTATTACAGGATGTTACTCCAGGAAACCCTGGTTACATTGAGGGAGAA
GCGTGATGCAGCTACGAGCTTACTGGCCGAGGAGCGAGAGAGAATGGTGACGATTCAGGAAGAAGAGCAGAGGTTTAAAGAGATGATCG
AGGCTGAGTATAAGATGCGATTCCAGTTACTGACTGAAGAGGAGGAACTGGCAGCCTACAAAGTTGACCTGAACTTAAATGGACCTAAT
CCAGACCAGCTGATGAAGTGTGGCACTGAGCTCATGCAGAAGTCCCAAGAAATGCTACAGAATGCAAAGTGCTATTTAAAAAGGAATGA
GTCTATACTGCTTCAGAGCCTAGAGCCAGCTCAGATAACAGACCTGAGTTCCTGCCAGATAATCGAAACGAGCCGTGTTCTACTGAGGC
TCCAAAGACATATTACTTTGGACCCAGGCACAGCTCACCCCTCCCTAGTCTTATCCGAAGACCTGAGAAGTGTGGGATTCCGAGAGACA
CCAAAGACTGGGCCTGGCACTACAAGGAGATTGGATTTCGGCGCTTCTGTGTTGGGTGCAGAGAGCTTCACCACGGGGAGGCATTATTG
GGAGGTGGCTGTGGGACAGGCAACCCAATGGCAGGTGGGCATATGTGACTGTACAGAGAGAAAGGGCAACATTCCTGGGGCTTCTGGAG
ATAAAGTCTTGCTCATGGGGTCCATGATGGGGACCGATTGTACCCTCTGGGTCTTTCCCCCTTTAAGAAAGGTCTGCCTGAGAAACCAA
ATGTACAAAGTTGGAGTCTTCCTAGACTGTGAATGTGGGCAAGTATCCTTCTACAACGTGACAGAGCAGTCCCTCATTTACAGTTTCTC
TGACCTTACCTTCCGAGGAGCCGTTAAACCAATATTTTCTCTTTGTATTCCAAATGGAGGCATGAGTTCAGACTCTCTCACTGTCTGTC
TTTCTCAGACTCATCCCTGAAATGCTGCTGATAGCTCTTTATCTTCCTGTGTGTGGAACCCAAGATCAAAAGAGACACAAAAATAAGAT
CCAGACTTTGTGAACATCACTTATGTAATTAATCTAATATAATCCAATAAAGCCATTAGACT

>TU7_isoform_A
TAGTTCCATGCTGTAGCTGAGGATGCTGGAGTGGTTTGTGTGTCCTGCTTTGTATGCTACTGCTGGGTGCTGTGAGGGGAATTCAGGCG
AGCTCTGACCTCGCGACATGCCTTCTATGTTTGAGAGAATCAGAGCTACACTGAGGAGCCTGTAAGACCTTCCTGCATGAAAATGATGC
ATTGACCTTTGATGATGTGCATGTACACTTCACTCGGGAGGAGTGGTCCTTGCTGGATCCATCTCAGAAGAGACTTTACAAAGATGTCA
TGCTGGAGAACTACAGGAATCTCACCACTATAGGCTACAATTGGAAAGATCATAACATTGAAGAACATTCTCAAAATGATAGAAGATAC
GGAAGGCACAAAATCACTCCTGCTGGAGAGAAACCCTATGAATATACACAATGGGATAAAGCCTTCAAATATCACAGTCATCTTCAAAA
GCTTGAAAGAAACCAAACTGGAGACAAAAACTCGGAAGTCTTTCAACATAGTAAAGTGCTTACATGTCAAAGTACTCTCCAAAGAAATA
AAAGAACAGATACTAGAGAGAAACCCTACAAATGTAATGAATGTGATAAAGCCTTTTTAAAGCTCAAGTATCTTCGACTACATGAAAGA
ATATATTCTGGGAAGAAACCCTATGAATGTAATCAATGTGGTAAAGCCTTTTTACATCCCAGCTATCTTCGAATGCATGAAAGAACACA
TACTGGAGAGAAACCTTACCAATGTAATCAATGTGGCAAAGCCTTTACACGAAGGAGTCATCTTCAAAGCCATAAAGTAGTACATACTG
GAGAGAGACCCTACATATGTAATCAGTGTAATAAAGCCTATCCACGCAAGAGTTGTCTCCGAAAACATGAAAGAACACATACTGGAGAG

AAACCTTACAAATGTGATCAGTGTGGTAAGGCCTTTACACAGAGCAGTCATCTTAAAACCCATAAAGTAATACATACTGGAGGGAGACT
CTAAATATGTAATCAGTGTGATGAAGCCTATTCACACCCGAGTCATCTTCCAAAACATGAAAGAACACATACTGGAGAGAAACCTTATA
AATAAATCAGTGTCATAAGTCATTTATATCTCACAGTCATCATCAAGCTGATGAAAGAATTTATACTGGATAGAAACCCTACAAATGTA
ATGAATGTGATAAAGCCTTTTCACAGCCCAGGTATCTTCGAATACATGAAAGAACACATACGGGAGAGAAACCTTACAAATGTTATCAG
TGTGATAAAGCATTTCCACAAGGCAATAATCTCAAAGTACATACAAGGAGGCATATTGGAGATATCCCTATGAATGAAATCAATGTGGT
AAAGCCTTTGTATATGACTAATCTTTTGGGATCCATAAAAGAACAGATAATGGAGACTGGAGAGAAACATTACCAACATAATCAATGTG
GTAAAGCCTTTGCAGAAAGCAGTCATCTTCAAACCCATAAAGTACTACATACTGGAGTGTAACCTCCC

>TU7_isoform_B
TAGTTCCATGCTGTAGCTGAGGATGCTGGAGTGGTTTGTGTGTCCTGCTTTGTATGCTACTGCTGGGTGCTGTGAGGGGAATTCAGGCG
AGCTCTGACCTCGCGACATGGATGCATTGACCTTTGATGATGTGCATGTACACTTCACTCGGGAGGAGTGGTCCTTGCTGGATCCATCT
CAGAAGAGACTTTACAAAGATGTCATGCTGGAGAACTACAGGAATCTCACCACTATAGGCTACAATTGGAAAGATCATAACATTGGAGA
ACATTCTCAAAATGATAGAAGATACGGAAGGCACAAAATCACTCCTGCTGGAGAGAAACCCTATGAATATACACAATGGGATAAAGCCT
TCAAATATCACAGTCATCTTCAAAAGCTTGAAAGAAACCAAACTGGAGACAAAAACTCGGAAGTCTTTCAACATAGTAAAGTGCTTACA
TGTCAAAGTACTCTCCAAAGAAATAAAAGAACAGATACTAGAGAGAAACCCTACAAATGTAATGAATGTGATAAAGCCTTTTTAAAGCT
CAAGTATCTTCGACTACATGAAAGAATATATTCTGGGAAGAAACCCTATGAATGTAATCAATGTGGTAAAGCCTTTTTACATCCCAGCT
ATCTTCGAATGCATGAAAGAACACATACCGGAGAGAAACCTTACCAATGTAATCAATGTGGCAAAGCCTTTACACGAAGGAGTCATCTT
CAAAGCCATAAAGTAGTACATACTGGAGAGAGACCCTACATATGTAATCAGTGTAATAAAGCCTATCCACGCAAGAGTTGTCTCCGAAA
ACATGAAAGAACACATACTGGAGAGAAACCTTACAAATGTGATCAGTGTGGTAAGGCCTTTACACAAAGCAGTCATCTTAAAACCCATA
AAGTAATACATACTGGAGGGAGACTCTAAATATGTAATCAGTGTGATGAAGCCTATTCACACCCGAGTCATCTTCCAAAACATGAAGGA
ACACATACTGGAGAGAAACCTTATAAATAAATCAGTGTCATAAGTCATTTATATCTCACAGTCATCATCAAGCTGATGAAAGAATTTAT
ACTGGATAGAAACCCTACAAATGTAATGAATGTGATAAAGCCTTTTCACAGCCCAGGTATCTTCGAATACATGAAAGAACACATACGGG
AGAGAAACCTTACAAATGTTATCAGTGTGATAAAGCATTTCCACAAGGCAATAATCTCAAAGTACATACAAGGAGGCATATTGGAGATA
TCCCTATGAATGAAATCAATGTGGTAAAGCCTTTGTATATGACTAATCTTTTGGGATCCATAAAAGAACAGATAATGGAGACTGGAGAG
AAACATTACCAACATAATCAATGTGGTAAAGCCTTTGCAGAAAGCAGTCATCTTCAAACCCATAAAGTACTACATACTGGAGTGTAACC
TCCC

>TU11_isoform_A
GCGGAAGCAAGTGAGAGATTTCGACCAGTTACCTTACTCTGGTTCTCCTACCTCCCTCTTCATCAGCGAGATTCAAGGCTGGACCAGGG
CCCCCAGGGAAAAAATCCTGCTATCAAGGAAGGAAATATAGGACCAGGACCTTCCCATGGACCCTTCTCAGGACGTTCCAACAGCACAA
GCAAGGGTGTCTCCCTAAGACACAAGAGGCCTGAAAACACTATCCTTTGTGAAGTGCCATGGTGGTCAGGAAAAGCAGAGCCACAGCTC
CCAGACTAAGCAAGCCTGGCCCCACAGGTACCTCACTTGGATCCCAGTGGGCGGGGCATCCCCACGTCCCCGCGCCATTAAGCCCAGGT
GGTGGACTCTACCCAAGGCACGGCCGGGGCAGAAGCGAAGGCCGGGAGGCGGGACTGAAGAATGCGCAGGCGGGAGCCACGAGGCGGGC
GCGCCCCTTTCACGGCTTCCGGCGGCGGCGGGCGGTTCCGGGGCGGCGGCGCGCGGCAGGGGACCGTTGGTGTTTGCGTTGGCCGTAGT
GGACTGGGCCGTGGACACCATGCAGCTGACCGTGAAGGCGCTCCAGGGCCGGGAATGTAGCCTACAGGTGGCGGAGGACGAGCTAGTGT
CTACACTGAAGCACCTGGTCTCGGATAAGCCGAATGTCCCTGTGCGCCAGCAACGTCTGCTGTTCAAGGGCAAGGCCCTAGCAGATGAA
AAACGACTGTCAGATTACAACATTGGGCCCAATTCTAAGCTCAACCTAGTTGTTAAGCCTTTGGAGAAGGTGCTACTGGAAGAAGGGTC
TGCCCACAGACTGGTCGACTCCCCAGCCACCCCCATCTGGCAGCTGATCTCCAAAGTCCTGGCCCGTCACTTCAGTGTAGCAGATGCCA
GCAGGGTCCTGGAACAACTACAGAGGGATTATGACAGGTCCTTGAGCCGCCTAACACTGGATGACATCGAACGTTTGGCCAGCCGCTTT
CTACACTCTGAAGTGACTGAGGCTATGGAAAAAGGGTTCTGCAAATAGCATTCTGGGAT

>TU11_isoform_B
GCGGAAGCAAGTGAGAGATTTCGACCAGTTACCTTACTCTGGTTTTCCTGCCTCCCTCTTCATCAGCGAGATTCAAGGCTGGACCAGGG
CCCCCAGGGAAAAAATCCTGCTATCAAGGAAGAAAATATAGGACCAGGACCTTCCCATGGACCCTTCTCAAGACATTCCAACAGCACAA
GCAAGGGTGTCTCCCTAAGACACAAGAGGCCTGAAAACACTATCCTTTGTGAAGTGCCATGGTGGTCAGGAAAAGCAGAGGTACCTCAC
TTGGATCCCAGTGGGCGGGGCATCCCCACGTCCCCGCGCCATTAAGCCCAGGTGGTGGACTCTACCCAAGGCACGGCCGGGGCAGAAGC
GAAGGCCGGGAGGCGGGACTGAAGAATGCGCAGGCGGGAGCCACGAGGCGGGCGCGCCCCTTTCACGGCTTCCGGCGGCGGCGGGCGGT
TCCGGGGCGGCGGCGCGCGGCAGGGGACCGTTGGTGTTTGCGTTGGCCGTAGTGGACTGGGCCGTGGACACCATGCAGCTGACCGTGAA
GGCGCTCCAGGGCCGGGAATGTAGCCTACAGGTGGCGGAGGACGAGCTAGTGTCTACACTGAAGCACCTGGTCTCGGATAAGCTGAATG
TCCCTGTGCGCCAGCAACGTCTGCTGTTCAAGGGCAAGGCCCTAGCAGATGAAAAACGACTGTCAGATTACAACATTGGGCCCAATTCT
AAGCTCAACCTAGTTGTTAAGCCTTTGGAGAAGGTGCTACTGGAAGAAGGGTCTGCCCACAGACTGGTCGACTCCCCAGCCACCCCCAT
CTGGCAGCTGATCTCCAAAGTCCTGGCCCGTCACTTCAGTGTAGCAGATGCCAGCAGGGTCCTGGAACAACTACAGAGGGATTATGACA
GGTCCTTGAGCCGCCTAACACTGGATGACATCGAACGTTTGGCCAGCCGCTTTCTACACTCTGAAGTGACTGAGGCTATGGAAAAAGGG
TTCTGCAAATAGCATTCTGGGAT

>TU11_isoform_C
GCGGAAGCAAGTGAGAGATTTCGACCAGTTACCTTACTCTGGTTTTCCTGCCTCCCTCTTCATCAGCGAGATTCAAGGCTGGACCAGGG
CCCCCAGGGAAAAAATCCTGCTATCAAGGAAGAAAATATAGGACCAGGACCTTCCCATGGACCCTTCTCAGGACATTCCAACAGCACAA
GCAAGGGTGTCTCCCTAAGACACAAGAGGCCTGAAAACACTATCCTTTGTGAAGTACCATGGATGTCAGGAAAAGCAGAGCCACAGCTC
CCAGACTAAGCAAGCCTGGCCCCACAGGTACCTCACTTGGATCCCAGTGGGCGGGGCATCCCCACGTCCCCGCGCCATTAAGCCCAGGT
GGTGGACTCTACCCAAGGCACGGCCGGGGCAGAAGCGAAGGCCGGGAGGCGGGACTGAAGAATGCGCAGGCGGGAGCCACGAGGCGGGC
GCGCCCCTTTCACGGCTTCCGGCGGCGGCGGGCGGTTCCGGGGCGGCGGCGCGCGGCAGGGGACCGTTGGTGTTTGCGTTGGCCGTAGT
GGACTGGGCCGTGGACACCATGCAGCTGACCGTGAAGGCGCTCCAGGGCCGGGAATGTAGCCTACAGGTGGCGGAGGACGAGCTAGTGT
CTACACTGAAGCACCTGGTCTCGGATAAGCTGAATGTCCCTGTGCGCCAGCAACGTCTGCTGTTCAAGGGCAAGGCCCTAGCAGGATTA
TGACAGGTCCTTGAGCCGCCTAACACTGGATGACATCGAACGTTTGGCCAGCCGCTTTCTACACCCTGAAGTGACTGAGGCTATGGAAA
AAGGGTTCTGCAAATAGCATTCTGGGAT

>Clk2-Scamp3_published_transcript
GTGGAACCGCACTTCGTCGCCGCGGGGGTGCCGGGACTCCGGCCGCAGTGCCGCCGCCATCACGGACTTCCTGTGGGACAAGCGCACGG
GCCTCGCCGCCAGAACGATGCCCCATCCCCGAAGGTACCATTCCTCAGAGCGAGGTAGCCGGGGGAGTTACCACGAACACTATCAGAGC
CGAAAGCATAAGCGAAGAAGAAGTCGCTCCTGGTCAAGTAGCAGTGACCGGACAAGGCGGCGGCGGAGGGAGGACAGCTACCACGTTCG
GTCCCGAAGCAGCTATGATGACCATTCGTCCGATCGGCGGCTGTACGATCGGCGGTACTGTGGCAGCTACAGGCGCAATGACTACAGCC

GGGACAGAGGGGAGGCTTACTACGACACAGACTTTCGGCAGTCCTATGAATACCATCGAGAGAACAGCAGTTACCGAAGCCAGCGCAGC
AGCCGAAGGAAACACAGAAGGCGGAGGAGACGGAGCCGGACATTCAGCCGCTCATCTTCACAGCACAGCAGCCGGAGAGCCAAGAGTGT
AGAGGACGACGCTGAGGGCCACCTCATCTACCACGTCGGGGACTGGCTACAAGAGCGATATGAAATTGTAAGCACCTTAGGAGAAGGGA
CCTTCGGCCGAGTTGTGCAGTGTGTGGACCATCGCAGGGGCGGAACACAAGTTGCCCTGAAGATCATTAAGAATGTGGAGAAGTACAAG
GAAGCAGCCCGACTAGAAATCAACGTGCTGGAGAAAATCAATGAGAAAGATCCTGACAACAAGAACCTCTGTGTCCAGATGTTTGACTG
GTTTGACTACCATGGCCACATGTGTATCTCCTTTGAGCTTCTGGGCCTTAGCACCTTCGATTTCCTCAAAGACAACAACTACCTGCCCT
ACCCCATCCACCAAGTGCGCCACATGGCCTTCCAGCTCTGCCAGGCCGTCAAGTTCCTCCATGATAACAAGTTGACACATACGGACCTC
AAACCTGAAAATATTCTGTTTGTGAATTCAGACTACGAACTCACCTACAACCTAGAGAAGAAGCGAGATGAGCGCAGTGTAAAGAGCAC
AGCCGTGCGGGTGGTGGACTTCGGCAGTGCCACCTTTGACCACGAACACCATAGCACCATTGTCTCCACTCGCCATTACCGAGCCCCCG
AGGTCATCCTGGAGTTGGGCTGGTCACAGCCATGCGATGTATGGAGCATAGGCTGCATCATCTTTGAGTACTACGTTGGCTTCACCCTC
TTCCAGACCCATGACAACAGAGAGCATCTGGCCATGATGGAAAGGATCCTGGGTCCTGTCCCTTCTCGGATGATCAGAAAGACAAGAAA
ACAGAAATATTTTTATCGGGGTCGCCTGGATTGGGATGAGAACACCTCAGCCGGGCGCTACGTTCGTGAGAACTGCAAACCTCTGCGGG
ACCCAGCTGTGATCCAGCACCGACCCAGCCAGCAGTATGCCACGCTGGACGTCTACAACCCCTTTGAGAACCGAGAGCCCCCACCAGCC
TACGAGCCTCCTGCTCCAGCCCCAGCACCTCTACCGCCACCCTCAGCTCCCTCTGTACAGTCCTCAAGAAAGCTCAGCCCCACAGAGCC
CAGGAACTACGGCTCCTACAGCACCCAGGCCTCAGCTGCAGCGGCCACTGCTGAGCTGCTGAAGAAGCAGGAGGAACTCAACCGCAAGG
CAGAGGAGTTGGACCGCCGGGAGCGAGAACTGCAGCATGTCGCCCTGGGTGGTGCAGGCACTCGACAGAACAACTGGCCTCCCCTACCG
TCTTTTTGCCCTGTAAAGCCTTGCTTTTTCCAAGACATCTCTATGGAGATACCCCAAGAATTTCAGAAGACGGTGTCCACCATGTACTA
CCTCTGGATGTGCAGCACTCTGGCTCTTCTCCTGAACTTCTTCGCCTGCCTGGCCAGGTTCTGTGTGGACACCGGCAGTGGCTCAGGCT
TTGGGCTGTCTATGCTCTGGCTGCTCCTTTTCACTCCCTGCTCCTTTGTCTGCTGGTACCGCCCGATGTATAAGGCTTTCCGGAGTGAT
AGTTCATTCAATTTCTTCGTTTTTTTCTTCATTTTCTTCGTCCAGGATGTGTTCTTTGTCCTCCAGGCCATTGGCATCCCAGGGTGGGG
CTTCAGCGGCTGGGTGACTGCTCTGGTGGTGGTGGGATCCAAACCTGCCGTGGCGGTGCTCATGCTGCTCGTCGCCCTGCTCTTCACCG
GCATCGCAGTGCTGGGAATCGTGATGCTGAAGCGGATCCACTCCCTGTACCGCCAAACGGGTGCCAGCTTTCAGAAGGCCCAGCAAGAA
TTTGCTGCTGGTGTCTTCTCCAACCCTGCGGTTCGAACTGCTGCTGCCAATGCAGCTGCCGGAGCTGCTGAAAATGCCTTCAGGGCCCC
TTGACCCCTGCTGCACCCGCTGGGATGCCCTGGGCCTGTCACCTGTGGGAACCAAGATAGTCCCTATCCCTGAAGTCTCTGGGATTCAG
GGAGCCATCACCACTTGGTGACTTCTATGGGGCTCTTCATTCCACAGCCATGACTTTAATCCTGACTTGAGGTGTGGAGAGCCCGCTGT
GACTCAGTCATTATTTGCCTTTTATCTCCCATCTGGCCACACTGCTGCCGCCACCTCTCACACAACCCCAACCCCAGTTTCCTTCTGCT
GTGCCAAGGATGTCTTC

>TU52_isoform_A
GTGGAACCGCACTTCGTCGCCGCGGGGGTGCCGGGACTCCGGCCGCAGTGCCGCCGCCATCACGGACTTCCTGTGGGACAAGCGCACGG
GCCTCGCCGCCAGAACGATGCCCCATCCCCGAAGGTACCATTCCTCAGAGCGAGGTAGCCGGGGGAGTTACCACGAACACTATCAGAGC
CGAAAGCATAAGCGAAGAAGAAGTCGCTCCTGGTCAAGTAGCAGTGACCGGACAAGGCGGCGGCGGAGGGAGGACAGCTACCACGTTCG
GTCCCGAAGCAGCTATGATGACCATTCGTCCGATCGGCGGCTGTACGATCGGCGGTACTGTGGCAGCTACAGGCGCAATGACTACAGCC
GGGACAGAGGGGAGGCTTACTACGACACAGACTTTCGGCAGTCCTATGAATACCATCGAGAGAACAGCAGTTACCGAAGCCAGCGCAGC
AGCCGAAGGAAACACAGAAGGCGGAGGAGACGGAGCCGGACATTCAGCCGCTCATCTTCAATGAAATTGTAAGCACCTTAGGAGAAGGG
ACCTTCGGCCGAGTTGTGCAGTGTGTGGACCGTCGCAGGGGCGGAACACGAGTTGCCCTGAAGATCATTAAGAATGTGGAGAAGTACAA
GGAAGCAGCCCGACTAGAAATCAACGTGCTGGAGAAAATCAATGAGAAAGATCCTGACAACAAGAACCTCTGTGTCCAGATGTTTGACT
GGTTTGACTACCATGGCCACATGTGTATCTCCTTTGAGCTTCTGGGCCTTAGCACCTTCGATTTCCTCAAAGACAACAACTACCTGCCC
TACCCCATCCACCAAGTGCGCCACATGGCCTTCCAGCTCTGCCAGGCCGTCAAGTTCCTCCATGATAACAAGTTGACACATACGGACCT
CAAACCTGAAAATATTCTGTTTGTGAATTCAGACTACGAACTCACCTACAACCTAGAGAAGAAGCGAGATGAGCGCAGTGTAAAGAGCA
CAGCCGTGCGGGTGGTGGACTTCGGCAGTGCCACCTTTGACCACGAACACCATAGCACCATTGTCTCCACTCGCCATTACCGAGCCCCA
GAGGTCATCCTGGAGTTGGGCTGGTCACAGCCCTGCGATGTATGGAGCATAGGCTGCATCATCTTTGAGTACTACGTTGGCTTCACCCT
CTTCCAGACCCATGACAACAGAGAGCATCTAGCCATGATGGAAAGGATCCTGGGTCCTGTCCCTTCTCGGATGATCAGAAAGACAAGAA
AACAGAAATATTTTTATCGGGGTCGCCTGGATTGGGATGAGAACACCTCAGCCGGGCGCTACGTTCGTGAGAACTGCAAACCTCTGCGG
GACCCAGCTGTGATCCAGCACCGACCCAGCCAGCAGTATGCCACGCTGGACGTCTACAACCCCTTTGAGAACCGAGAGCCCCCACCAGC
CTACGAGCCTCCTGCTCCAGCCCCAGCACCTCTACCGCCACCCTCAGCTCCCTCTGTACAGTCCTCAAGAAAGCTCAGCCCCACAGAGC
CCAGGAACTACGGCTCCTACAGCACCCAGGCCTCAGCTGCAGCGGCCACTGCTGAGCTGCTGAAGAAGCAGGAGGAACTCAACCGCAAG
GCAGGGGAGTTGGACCGCCGGGAGCGAGAACTGCAGCATGTCGCCCTGGGTGGTGCAGGCACTCGACAGAACAACTGGCCTCCCCTACC
GTCTTTTTGCCCTGTAAAGCCTTGCTTTTTCCAAGACATCTCTATGGAGATACCCCAAGAATTTCAGAAGACGGTGTCCACCATGTACT
ACCTCTGGATGTGCAGCACTCTGGCTCTTCTCCTGAACTTCTTCGCCTGCCTGGCCAGGTTCTGTGTGGACACCGGCAGTGGCTCAGGC
TTTGGGCTGTCTATGCTCTGGCTGCTCCTTTTCACTCCCTGCTCCTTTGTCTGCTAGTACCGCCCGATGTATAAGGCTTTCCGGAGTGA
TAGTTCATTCAATTTCTTCGTTTTTTTCTTCATTTTCTTCGTCCAGGATGTGTTCTTTGTCCTCCAGGCCATTGGCATCCCAGGGTGGG
GCTTCAGCGGCTGGGTGACTGCTCTGGTGGTGGTGGGATCCAAACCTGCCGTGGCGGTGCTCATGCTGCTCGTCGCCCTGCTCTTCACC
GGCATCGCAGTGCTGGGAATCGTGATGCTGAAGCGGATCCACTCCCTGTACCGCCAAACGGGTGCCAGCTTTCAGAAGGCCCAGCAAGA
ATTTGCTGCTGGTGTCTTCTCCAACCCTGCGGTTCGAACTGCTGCTGCCAATGCAGCTGCCGGAGCTGCTGAAAATGCCTTCAGGGCCC
CTTGACCCCTGCTGCACCCGCTGGGATGCCCTGGGCCTGTCACCTGTGGGAACCAAGATAGTCCCTATCCCTGAAGTCTCTGGGATTCA
GGGAGCCATCACCACTTGGTGACTTCTATGGGGCTCTTCATTCCACAGCCATGACTTTAATCCTGACTTGAGGTGTGGAGAGCCCGCTG
TGACTCAGTCATTATTTGCCTTTTATCTCCCATCTGGCCACACTGCTGCCGCCACCTCTCACACAACCCCAACCCCAGTTTCCTTCTGC
TGTGCCAAGGATGTCTTC

>TU52_isoform_B
GTGGAACCGCACTTCGTCGCCGCGGGGGTGCCGGGACTCCGGCCGCAGTGCCGCCGCCATCACGGACTTCCTGTGGGACAAGCGCACGG
GCCTCGCCGCCAGAACGATGCCCCATCCCCGAAGGTACCATTCCTCAGAGCGAGGTAGCCGGGGGAGTTACCACGAACACTATCAGAGC
CGAAAGCATAAGCGAAGAAGAAGTCGCTCCTGGTCAAGTAGCAGTGACCGGACAAGGCGGCGGCGGAGGGAGGACAGCTACCACGTTCG
GTCCCGAAGCTATGATGACCATTCGTCCGATCGGCGGCTGTACGATCGGCGGTACTGTGGCAGCTACAGGCGCAATGGCTACAGCCGGG
ACAGAGGGGAGGCTTACTACGACACAGACTTTCGGCAGTCCTATGAATACCATCGAGAGAACAGCAGTTACCGAAGCCAGCGCAGCAGC
CGAAGGAAACACAGAAGGCGGAGGAGACGGAGCCGGACATTCAGCCGCTCATCTTCACACAGCAGCCGGAGAGCCAAGAGTGTAGAGGA
CGACGCTGAGGGCCACCTCATCTACCACGTCGGGGACTGGCTACAAGAGCGATATGAAATTGTAAGCACCTTAGGAGAAGGGACCTTCG
GCCGAGTTGTGCAGTGTGTGGACCATCGCAGGGGCGGAACACAAGTTGCCCTGAAGATCATTAAGAATGTGGAGAAGTACAAGGAAGCA
GCCCGACTAGAAATCAACGTGCTGGAGAAAATCAATGAGAAAGATCCTGACAACCAGAACCTCTGTGTCCAGATGTTTGACTGGTTTGA
CTACCATGGCCCCATGTGTATCTCCTTTGAGCTTCTGGGGCTTAGCACCTTCGATTTCCTCAAAGACAACAACTACCTGCCCTACCCCA
TCCACCAAGTGCGCCACATGGCCTTCCAGCTCTGCCAGGCCGTCAAGTTCCTCCATGATAACAAGTTGACACATACGGACCTCAAACCT

GAAAATATTCTGTTTGTGAATTCAGACTACGAACTCACCTACAACCTAGAGAAGAAGCGAGATGAGCGCAGTGTAAAGAGCACAGCCGT
GCGGGTGGTGGACTTCGGCAGTGCCACCTTTGACCACGAACACCATAGCACCATTGTCTCCACTCGCCATTACCGAGCCCCCGAGGTCA
TCCTGGAGTTGGGCTGGTCACAGCCATGCGATGTATGGAGCATAGGCTGCATCATCTTTGAGTACTGCGTTGGCTTCACCCTCTTCCAG
ACCCATGACAACAGAGAGCATCTAGCCATGATGGAAAGGATCCTGGGTCCTGTCCCTTCTCGGATGATCAGAAAGACAAGAAAACAGAA
ATATTTTTATCGGGGTCGCCTGGATTGGGATGAGAACACCTCAGCCGGGCGCTACGTTCGTGAGAACTGCAAACCTCTGCGGCGGCTGG
GTGACTGCTCTGGTGGTGGTGGGATCCAAACCTGCCGTGGCGGTGCTCATGCTGCTCGTCGCCCTGCTCTTCACCGGCATCGCAGTGCT
GGGAATCGTGATGCTGAAGCGGATCCACTCCCTGTACCGCCAAACGGGTGCCAGCTTTCAGAAGGCCCAGCAAGAATTTGCTGCTGGTG
TCTTCTCCAACCCTGCGGTTCGAACTGCTGCTGCCAATGCAGCTGCCGGAGCTGCTGAAAATGCCTTCAGGGCCCCTTGACCCCTGCTG
CACCCGCTGGGATGCCCTGGGCCTGTCACCTGTGGGAACCAAGATAGTCCCTATCCCTGAAGTCTCTGGGATTCAGGGAGCCATCACCA
CTTGGTGACTTCTATGGGGCTCTTCATTCCACAGCCATGACTTTAATCCTGACTTGAGGTGTGGAGAGCCCGCTGTGACTCAGTCATTA
TTTGCCTTTTATCTCCCATCTGGCCACACTGCTGCCGCCACCTCTCACACAACCCCAACCCCAGTTTCCTTCTGCTGTGCCAAGGATGT
CTTC

>TU54
AGATGGAACCGGAGCTGACCACGGGGAGAAGAGCGGTGTGGGAGCCCCAGACCCCGGGGCTGCACACGCTGAAGGGGAGCTCGTTCCCG
AACCGGAGCAGGACTTCGGAGGGGACTCGGTGGCACCTCTAGACCTCGGGAAAGGGACTTCTCCTGGGGACCGAAGCCTCCCTTACTCT
GCTTTCCCGGGCGGCGACACCGAATCCCTGTGCGGAGACCCCGGTGTCCAGACGAATGAGGAGCAGGAGAGGACCCGCCACGACACCGC
GGGCTCAGCCATGGGTGACCCCGGAACCGAGAGCATCGCACCCGTGTGCCCTCCTTTACAGTGTAATCAGGACGAAGACTCCTTGACTT
TGCTCATCCAAGTGCCTGGGATTCTGCCACAAACTCTTCACGGACATCTAAGCCCCGTCGGTTACGAATTGTGCTTCTCTACACAAGAC
TCGGGTTATTCCTGCACTTTGCAGTTTGCTCCAGAGAATAAGTTGAGTACCAGAGAACCCGAGACTAGCGTTTCTTTAAATAATGCAGT
CATAGTACTGGCAAAATCTCCAGAGAGCCACGGACTTTGGCGAGAGTGGTATTGTGGCTTGAACAAGGAGTCTTTGGAGGAAAGATTGT
TTATCAATGAAGAAAATGTTAATGGGTTTCTTGAAGAGGTCCTTTGCTCCCCATTGAAGCAGGCAAGGTCTCTGGCCCCACCGCTGATT
GAGGTTCTTCAGGCCACTGATGAGCAGGTTCAGATCCATGCAGAGGTGGGGAAATGGCTGCCATAGGAAACTTCTCACCGATAACTAGA
GTTTCCGGAAACAGTTCTCATCGCAGACACTTCTACTGCAAACATGGTCAACGTACCAAAAACCCGAAGGACTTTCTGTAAGAAATGTG
GCAAGCATCAGCCTCACAAAGTGACCCAGTATAAGAAGGGCAAGGATTCCCTGTATGCCCAAGGAAAGCGGCGCTACGATCGCAAGCAG
AGTGGCTACGGTGGGCAGACAAAGCCAATCTTTCGAAAGAAGGCCAAAACCACAAAGAAGATTGTGCTCAGGCTTGAGTGTGTTGAGCC
CAACTGCAGATCCAAGAGGATGCTGGCCATTAAGAGATGCAAGCATTTTGAACTGGGAGGCGACAAGAAGAGGAAGGGC
