# Supplementary material for: Detailed characterization of the mouse embryonic stem cell transcriptome reveals novel genes and intergenic splicing associated with pluripotency
Source: BMC Genomics. 2008 Apr 9;9:155. doi: 10.1186/1471-2164-9-155 (PMC2375908; doi:10.1186/1471-2164-9-155)
Supplement: Additional file 3 — Oct4, Nanog, and Sox2 binding sites in the 100 kb vicinity of the TUs identified from chromatin immunoprecipitation paired-end diTags (ChIP-PET) [3] and ChIP-seq [H. H. Ng et al., unpublished]. [file 1471-2164-9-155-S3.pdf]

TU	Nearest Oct4 ChIP-seq (unpublished)	Nearest Oct4 ChIP-PET (Loh et al. 2006)	Nearest Nanog ChIP-seq (unpublished)	Nearest Nanog ChIP-PET (Loh et al. 2006)	Nearest Sox2 ChIP-seq (unpublished)	Nearest Sox2 ChIP-PET (unpublished)
4	none	none	at TSS	none	none	none
7	~27.5 kb downstream	~ 45 kb upstream	~27.5 kb downstream	none	none	none
11	none	none	~62.5 kb downstream	none	none	none
52	intragenic	~ 65 kb downstream	at end of transcript	none	~42.5 kb downstream	~42.5 kb downstream
54	~15 kb downstream	none	>5 kb upstream	none	none	none

Oct4, Nanog, and Sox2 binding sites in the vicinity of the five novel TUs selected for follow-up analysis, as assessed by ChIP-PET and ChIP-sequencing (Solexa) approaches
