# Supplementary figures and images for: Detailed characterization of the mouse embryonic stem cell transcriptome reveals novel genes and intergenic splicing associated with pluripotency
Source: BMC Genomics. 2008 Apr 9;9:155. doi: 10.1186/1471-2164-9-155 (PMC2375908; doi:10.1186/1471-2164-9-155)

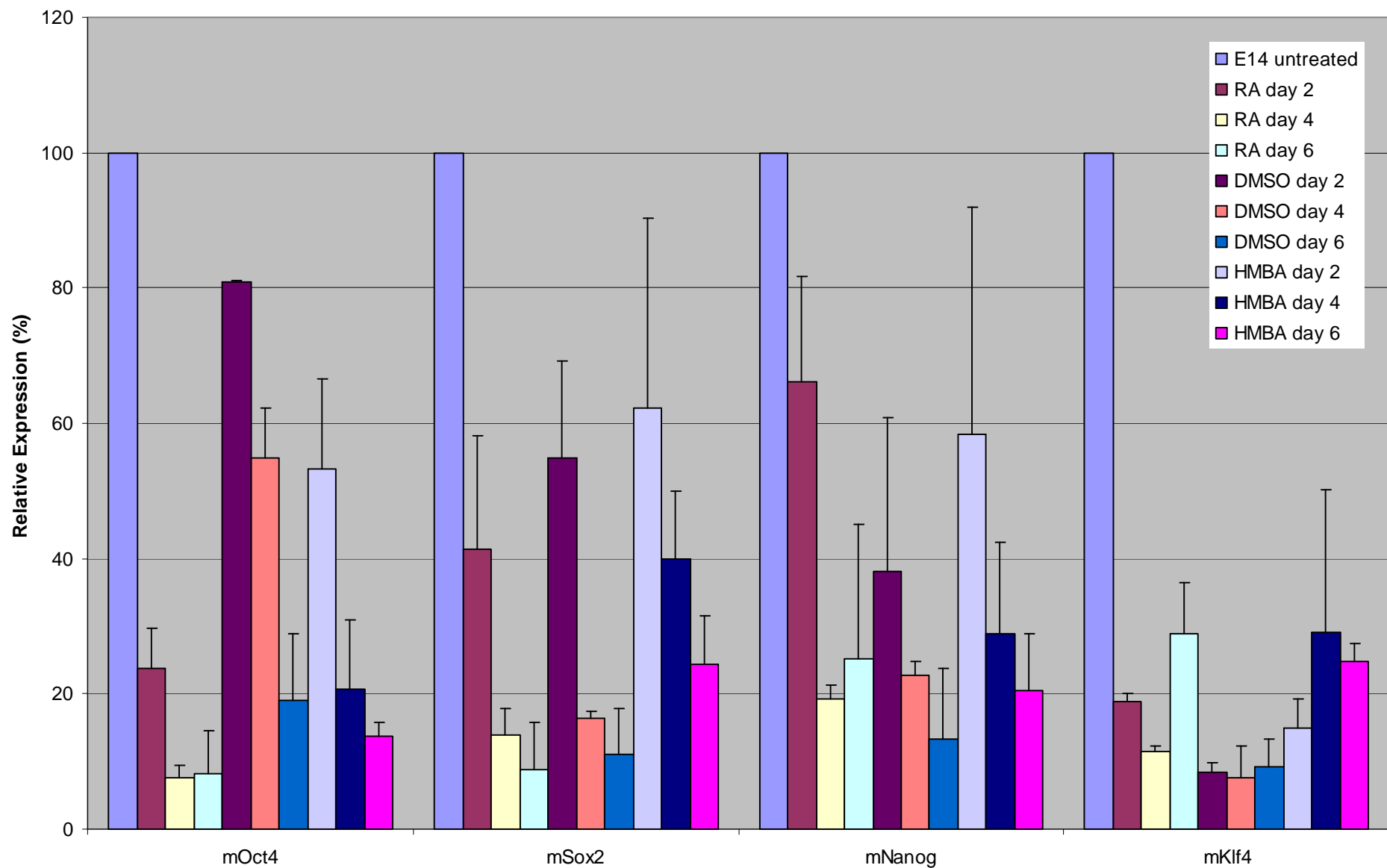

Supplement: Additional file 4 — Expression levels of the pluripotency markers Oct4, Sox2, Nanog, and Klf4 upon RA-, DMSO-, and HMBA-induced differentiation. [file 1471-2164-9-155-S4.pdf]
