# Supplementary material for: Detailed characterization of the mouse embryonic stem cell transcriptome reveals novel genes and intergenic splicing associated with pluripotency
Source: BMC Genomics. 2008 Apr 9;9:155. doi: 10.1186/1471-2164-9-155 (PMC2375908; doi:10.1186/1471-2164-9-155)
Supplement: Additional file 5 — Primer sequences used to characterize the TUs identified in this study. [file 1471-2164-9-155-S5.pdf]

Name	Primer sequence	Tm/ ºC	Remarks
TU2	ACTTAGTAACATTCCCTCAGCC	56.6	Forward
	GACGTGAGCAGTCTCCAGAAA	60.6	Reverse
TU3	GCAGCAGCACTCACCCACTGAG	59.5	Forward
	GGGAGGAAACTTTCCACTCCTAG	54.3	Reverse
TU4	GTACTGAAGTCTCTGGTCTCC	52.9	Forward
	AGTCTAATGGCTTTATTGGATTA	54.2	Reverse
	AGCTCATGCAGAAGTCCCAAG	61.9	Real Time PCR Forward
	TGCTCACCAAGCCAGACTTC	61.6	Real Time PCR Reverse
TU6	GGCTCAGGCTGTCTGAAAAG	60.1	Forward
	TGGTCAATTTCTGAAGGTATGC	59.1	Reverse
	GCTGTCTGAAAAGATGGAAAGG	60.2	Nested Forward
	ATTTCTGAAGGTATGCTACACAATAG	57.3	Nested Reverse
TU7	TAGTTCCATGCTGTAGCTGAGG	59.5	Forward
	GGGAGGTTACACTCCAGTATG	56.1	Reverse
	TGTCCTGCTTTGTATGCTACTGC	61.7	Real Time PCR Forward
	CCAGCATGACATCTTTGTAAAGTC	60.1	Real Time PCR Reverse
TU8	GCTGGCTATAAGTTCTCGAAGGC	55.5	Forward
	CAGCTCCAGGTCCGCAGGGATC	63.7	Reverse
TU9	AGATGTTTTCTTACCGCTCTG	56.3	Forward
	TTGTTTTAATGCGTCTTTCACTC	51	Reverse
	GTCCACGCCTGTAAAATTGG	60.4	Nested Forward
TU10	GTCGGGAATACAGAAGAAAGAAT	50.4	Forward
	GTGGCTTAGATTATCTGCACG	49.5	Reverse
	TCCCTGCGAGTACACCTCTC	60.4	Nested Forward
TU11	GCGGAAGCAAGTGAGAGATTTC	54.7	Forward
	ATCCCAGAATGCTATTTGCAG	51.2	Reverse
	AGCAAGTGAGAGATTTCGACC	50.2	Nested Forward
	TTTCCATAGCCTCAGTCACTTC	50.4	Nested Reverse
	GTGCCATGGTGGTCAAGAAAAG	60.5	TaqMan Forward
	GGCCAGGCTTGCTTAGTCT	59.6	TaqMan Reverse
	CAGAGCCACAGCTCC	50.8	TaqMan Probe
TU12	GACGCTGGGTCAAGATCGCCAC	62.5	Forward
	TCGGTGTCCTTACGCTTTATTT	52.6	Reverse
TU15	AGTAGTGCCCTGAGGCGCCAG	60.2	Forward
	TAGTTGAAGATACTTTAGTGATGGAAATTTC	54.9	Reverse
TU17	GCTGGCTTTAGACAAAGCGGC	57.9	Forward
	CAACACAAGGGTGACAAAGGA	52.4	Reverse
	GCCACCGCCGCTCATCTGAAG	63.5	Nested Forward
	AACCACATCCTCTCTGTCGGG	55.2	Nested Reverse

TU52	GTGGAACCGCACTTCGTC	60.3	Forward
	GAAGACATCCTTGGCACAGC	60.8	Reverse
	CGCTACGTTCGTGAGAACTG	59.7	Real Time PCR Forward
	GGCTGAGCTTTCTTGAGGAC	59.2	Real Time PCR Reverse
TU53	ACTCTTCCGCACCCTGCTC	62.9	Forward
	GAAGAAGTTTGGAGTTCTTTATTGC	59	Reverse
TU54	AGATGGAACCGGAGCTGAC	60.2	Forward
	GCCCTTCCTCTTCTTGTCG	59.9	Reverse
	GCCACTGATGAGCAGGTTCAG	63.3	Real Time PCR Forward
	CGTTGACCATGTTTGCAGTAG	59.3	Real Time PCR Reverse
TU55	CAGATTTCCTGTGGAGTAAGGAC	59.2	Forward
	ACCGACTAGGTTGGGTTGAC	58.9	Reverse
ActB	TACAGCTTCACCACCACAGC	59.9	Real Time PCR Forward
	AGTTTCATGGATGCCACAGG	60.9	Real Time PCR Reverse
Slc10a3	CTTCATCAGCGAGATTCAAGG	60	Real Time PCR Forward
	ACTGCTGGGAGCTACCTCTG	59.6	Real Time PCR Reverse
Ubl4	TGGTCTCGGATAAGCTGAATG	60.2	Real Time PCR Forward
	CCAGGACTTTGGAGATCAGC	59.8	Real Time PCR Reverse
M13-F	GTAAAACGACGGCCAG	50	Forward
M13-R	CAGGAAACAGCTATGAC	50	Reverse

Primer sequences used to characterize the TUs identified in this study
