# Supplementary material for: Detailed characterization of the mouse embryonic stem cell transcriptome reveals novel genes and intergenic splicing associated with pluripotency
Source: BMC Genomics. 2008 Apr 9;9:155. doi: 10.1186/1471-2164-9-155 (PMC2375908; doi:10.1186/1471-2164-9-155)
Supplement: Additional file 6 — Catalog numbers of mouse total RNA (BD Biosciences Clontech) used in the multi-tissue panel expression analysis. [file 1471-2164-9-155-S6.pdf]

Tissue Type	Catalog No.
7-day Embryo	636607
11-day Embryo	636608
15-day Embryo	636609
17-day Embryo	636610
Brain (whole)	636601
Colon	636669
Heart	636602
Kidney	636612
Liver	636603
Lung	636604
Ovary	636671
Skeletal Muscle	636673
Spleen	636605
Stomach	636617
Testis	636606
Thymus	636618

Catalog numbers of mouse total RNA (BD Biosciences Clontech) used in the multi-tissue panel expression analysis
