# Supplementary material for: Detailed characterization of the mouse embryonic stem cell transcriptome reveals novel genes and intergenic splicing associated with pluripotency
Source: BMC Genomics. 2008 Apr 9;9:155. doi: 10.1186/1471-2164-9-155 (PMC2375908; doi:10.1186/1471-2164-9-155)
Supplement: Additional file 7 — Catalog numbers of TaqMan probes (Applied Biosystems) used in the QRTPCR. [file 1471-2164-9-155-S7.pdf]

Gene Symbol	Lineage	ABI TaqMan Gene Expression Assay ID
Sox17	Endodermal	Mm00488363_m1
Gata4	Endodermal	Mm00484689_m1
Gata6	Endodermal	Mm00802636_m1
Foxa2	Endodermal	Mm00839704_mH
Afp	Endodermal	Mm00431715_m1
Gata2	Mesodermal	Mm00492300_m1
Nkx2.5	Mesodermal	Mm00657783_m1
T-brachyury	Mesodermal	Mm00436876_g1
MyoD	Mesodermal	Mm00440387_m1
Hand1	Mesodermal	Mm00433931_m1
Bmp4	Mesodermal	Mm00432087_m1
Fgf5	Ectodermal	Mm00438919_m1
Nestin	Ectodermal	Mm00450205_m1
Sox1	Ectodermal	Mm00486299_s1
Sox4	Ectodermal	Mm00486317_s1
Pax6	Ectodermal	Mm00443072_m1
Pax7	Ectodermal	Mm00834079_m1
Rest	Ectodermal	Mm00803268_m1
Oct4	Pluripotency	Mm00658129_gH
Sox2	Pluripotency	Mm00488369_s1
Nanog	Pluripotency	Mm01617762_g1
Klf4	Pluripotency	Mm00516104_m1
ActB	Control	Mm00607939_s1

Catalog numbers of TaqMan probes (Applied Biosystems) used in the QRTPCR
