# Supplementary material for: Detailed characterization of the mouse embryonic stem cell transcriptome reveals novel genes and intergenic splicing associated with pluripotency
Source: BMC Genomics. 2008 Apr 9;9:155. doi: 10.1186/1471-2164-9-155 (PMC2375908; doi:10.1186/1471-2164-9-155)
Supplement: Additional file 8 — Sequences of the custom siRNA (Dharmacon) designed for the TUs. [file 1471-2164-9-155-S8.pdf]

Target	siRNA Target Sequences
TU4	CGAAACGAGCCGUGUUCUA
	CUUCCGAGGAGCCGUUAAA
	AGGAGCCGUUAAACCAAUA
	GGAGCCGUUAAACCAAUAU
TU7	CAGUGUCAUAAGUCAUUUA
	CGACUACAUGAAAGAAUAU
	GAAUGAAAUCAAUGUGGUA
	GGAAAGAUCAUAACAUUGA
TU11	GCCCAGGUGGUGGACUCUA
	GCCACAGCUCCCAGACUAA
TU52	ACCUCUGCGGGACCCAGCU
TU54	GGAAACAGUUCUCAUCGCA

Sequences of the custom siRNA (Dharmacon) designed against the TUs
